# Sialylated glycoproteins suppress immune cell killing by binding to Siglec-7 and Siglec-9 in prostate cancer

**DOI:** 10.1172/JCI180282

**Published:** 2024-10-22

**Authors:** Ru M. Wen, Jessica C. Stark, G. Edward W. Marti, Zenghua Fan, Aram Lyu, Fernando Jose Garcia Marques, Xiangyue Zhang, Nicholas M. Riley, Sarah M. Totten, Abel Bermudez, Rosalie Nolley, Hongjuan Zhao, Lawrence Fong, Edgar G. Engleman, Sharon J. Pitteri, Carolyn R. Bertozzi, James D. Brooks

**Affiliations:** 1 Department of Urology,; 2 Department of Chemistry, and Sarafan ChEM-H, Stanford University, Stanford, California, USA.; 3 Department of Biological Engineering, Department of Chemical Engineering, and Koch Institute for Integrative Cancer Research, Massachusetts Institute of Technology, Cambridge, Massachusetts, USA.; 4 Department of Molecular and Cellular Physiology, Stanford University, Stanford, California, USA.; 5 Department of Medicine, UCSF, San Francisco, California, USA.; 6 Parker Institute for Cancer Immunotherapy, San Francisco, California, USA.; 7 Department of Radiology,; 8 Canary Center at Stanford for Cancer Early Detection, and; 9 Department of Pathology, Stanford University, Stanford, California, USA.; 10 Howard Hughes Medical Institute, Stanford, California, USA.

**Keywords:** Oncology, Cancer immunotherapy, Immunotherapy, Prostate cancer

## Abstract

Prostate cancer is the second leading cause of male cancer death in the U.S. Current immune checkpoint inhibitor–based immunotherapies have improved survival for many malignancies; however, they have failed to prolong survival for prostate cancer. Siglecs (sialic acid–binding immunoglobulin-like lectins) are expressed on immune cells and regulate their function. Siglec-7 and Siglec-9 contribute to immune evasion in cancer by interacting with sialic acid–containing glycoprotein ligands on cancer cells. However, the role of Siglec-7/9 receptors and their ligands in prostate cancer remains poorly understood. Here, we find that Siglec-7 and Siglec-9 are associated with poor prognosis in patients with prostate cancer and are highly expressed in myeloid cells, including macrophages, in prostate tumor tissues. Siglec-7 and -9 ligands were expressed in prostate cancer cells and human prostate tumor tissues. Blocking the interactions between Siglec-7/9 and sialic acids inhibited prostate cancer xenograft growth and increased immune cell infiltration in humanized mice in vivo. Using a CRISPRi screen and mass spectrometry, we identified CD59 as a candidate Siglec-9 ligand in prostate cancer. The identification of Siglec-7 and -9 as potential therapeutic targets, including the CD59/Siglec-9 axis, opens up opportunities for immune-based interventions in prostate cancer.

## Abstract 2

Checkpoint inhibitors have limited effectiveness in prostate cancer. Siglecs-7 and -9 contribute to immune evasion and blocking Siglec-7/9-sialic acid interactions suppresses tumor progression, indicating therapeutic potential.

## Graphical abstract

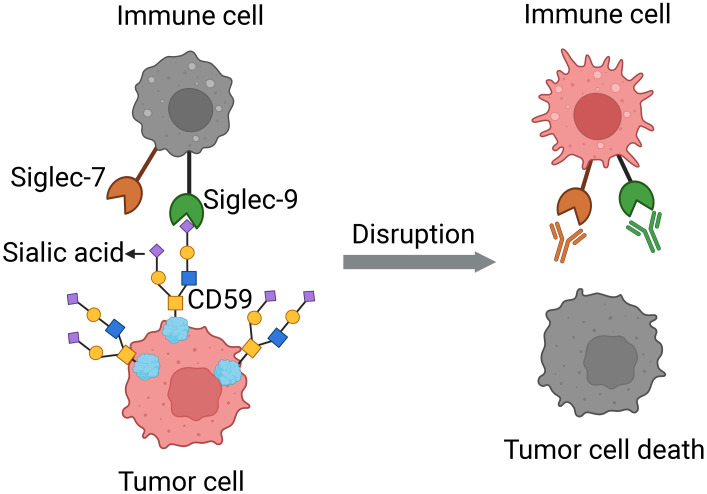

## Introduction

Prostate cancer (PCa) is one of the most prevalent cancers in men worldwide, with a 5-year survival rate of under 30% for men with metastatic disease, despite treatment with androgen deprivation therapies (1–3). While immune checkpoint inhibitor–based (ICI-based) immunotherapies have quickly become the standard of care for several malignancies (4, 5), they have been ineffective in treating PCa, except for a small subset (approximately 1%) with mismatch repair gene mutations (6–9). However, dendritic cell therapies, such as sipuleucel-T, can prolong survival in many advanced PCas and suggest that alternative immune checkpoint pathways could be utilized during disease development and progression (10, 11).

Siglecs, sialic acid–binding immunoglobulin-like lectins, are a family of glycan-binding immunoreceptors that recognize glycans or glycoconjugates bearing the sialic acid monosaccharide (12). The Siglec family is composed of both activating and inhibitory receptors, which each bind discrete sialoglycan ligands. The majority of CD33-related Siglecs function as inhibitory receptors, including Siglec-5, Siglec-8, Siglec-7, Siglec-9, and Siglec-10 (13–16). Siglec-7 and Siglec-9 are expressed by NK cells, monocytes, dendritic cells (DCs), macrophages, and subsets of T cells in the tumor microenvironment (17–22), and a growing body of evidence implicates these Siglecs as potential immune checkpoints in cancer (23–28).

Aberrant expression of sialic acid–containing glycans (sialoglycans) by cancer cells was initially observed in 1960s (29). More recently, these sialoglycans have been identified as Siglec ligands that can bind directly to Siglec receptors on immune cells in the tumor microenvironment and cause immune suppression (27, 30–32). For example, sialylated glycans on the surface of melanoma, lung cancer, and pancreatic cancer cells have been shown to bind to Siglec-7/9 on immune cells to inhibit the immune response (20, 33, 34). Previous work has demonstrated that cancer cell lines PC3 and DU145 express ligands for Siglec-7 and -9 (35, 36); however, the role of the Siglec-7 and -9–sialic acid pathway in PCa remains poorly understood.

To better understand whether Siglec-7 and -9 could have a role in immune-suppressive pathways in PCa, we analyzed the expression profiles of Siglec isoforms in patient samples and examined their correlation with clinicopathological parameters. We also examined the expression levels of Siglec-7 and Siglec-9 ligands in PCa cells and tumors and interrogated their functional effects in vivo. Finally, we used discovery-based approaches to identify candidate Siglec ligands expressed by PCa cells and evaluated their potential as therapeutic targets.

## Results

### Elevated Siglec-7/9 expression is correlated with higher recurrence rates in patients with PCa.

**Figure 1:**
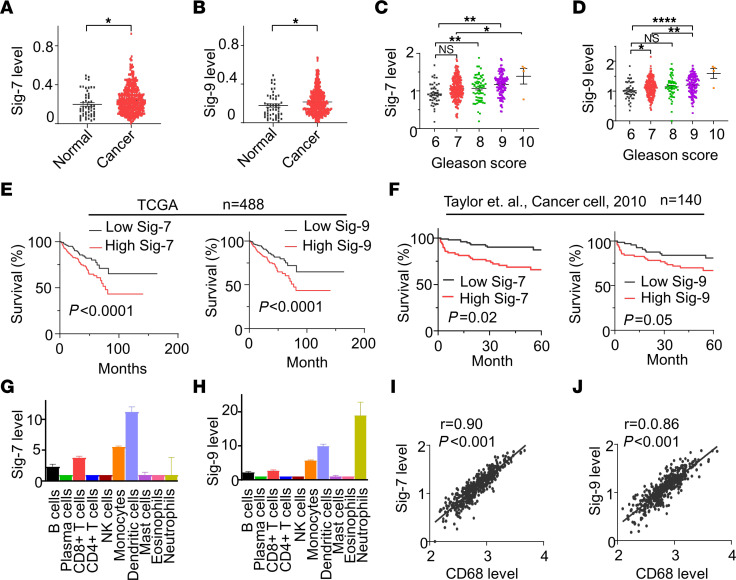
High Siglec-7/9 expression is correlated with worse clinical outcome in patients with PCa. Violin plots showing that (**A**) Siglec-7 and (**B**) Siglec-9 mRNA expression are significantly higher in tumor tissues (*n* = 497) than normal tissues (*n* = 53). Data were analyzed by unpaired student’s *t* test. (**C**) Siglec-7, and (**D**) Siglec-9 are correlated with Gleason Score. Data were analyzed by 1-way ANOVA with post hoc Tukey’s test and presented as mean ± SEM. (**E**) High Siglec-7 and Siglec-9 expression is correlated with worse survival in patients with PCa in the TCGA-PRAD database (*n* = 488). (**F**) High Siglec-7 and Siglec-9 expression are correlated with worse survival in patients with PCa in MSKCC database (*n* = 140). Survival analysis was conducted using the Kaplan–Meier (log-rank test). Median expression was used as cutoff between high and low Siglec-7 and -9 expressing groups. (**G**) Siglec-7, and (**H**) Siglec-9 are highly expressed in monocyte, dendritic cells, and CD8^+^ T cells identified by CIBERSORTx from the TCGA-PRAD database. (**I** and **J**) Scatter plot of RNA expression of (**I**) Siglec-7 and (**J**) Siglec-9 compared with CD68 in prostate tumor tissues (*n* = 494), according to TCGA data. Siglec-7, and Siglec-9 are correlated with macrophage marker CD68. Data were analyzed by Pearson’s correlation. *P ≤ 0.05; **P ≤ 0.01; ***P ≤ 0.0001.

To determine the association of Siglec-7 and Siglec-9 expression with recurrence-free survival (RFS) in PCa, we assessed the association of Siglec expression with clinical outcomes in publicly available datasets. In the Cancer Genome Atlas–Prostate Adenocarcinoma (TCGA-PRAD) dataset, transcript levels of Siglec-1, -6, -7, -9, -15, and -16 were significantly higher in tumor samples compared with normal tissues (Figure 1, A and B and Supplemental Figure 1; supplemental material available online with this article; https://doi.org/10.1172/JCI180282DS1). Increased expression levels of Siglec-1, Siglec-7, and Siglec-9 were associated with higher cancer grade (Gleason scores) in PCa (Figure 1, C and D and Supplemental Figure 2). Moreover, the expression levels of Siglec-7 and Siglec-9 showed a significant association with RFS in 488 PCa patients (Figure 1E). Patients with high expression levels of Siglec-7 and Siglec-9 also exhibited worse RFS outcomes in a cohort of 140 patients with PCa in the Memorial Sloan Kettering Cancer Center (MSKCC)dataset (37) (Figure 1F), suggesting Siglec-7 and Siglec-9 may play a promoting role in PCa progression.

To gain insights into the immune cell populations associated with Siglec expression and clinical outcomes, CIBERSORTx, an analytical tool employing machine learning techniques, was utilized to deconvolute the composition of cell populations based on gene expression data (38). CIBERSORTx analysis of TCGA-PRAD dataset showed that tumor infiltrating immune cells expressed different Siglec profiles in PCa (Supplemental Figure 3). Specifically, DCs, monocytes, and CD8^+^ T cells were predominantly associated with the expression of Siglec-7 and Siglec-9, while tumor infiltrating neutrophils expressed high levels of Siglec-9 (Figure 1, G and H). Due to the limitations of immune cell type definitions by the CIBERSORTx algorithm, Siglec-7 and Siglec-9 expression in certain immune cells, including macrophages, could not be identified. Therefore, we performed a Pearson correlation analysis between Siglec-7 and Siglec-9 expression and a macrophage marker and found that both are correlated with the macrophage marker CD68 (Figure 1, I and J). These results suggest that Siglec-7 and Siglec-9 may play an important role in immune cell functions in the PCa tumor environment.

### High Siglec-7/9 expression is found in myeloid cells in human PCa tumors.

**Figure 2:**
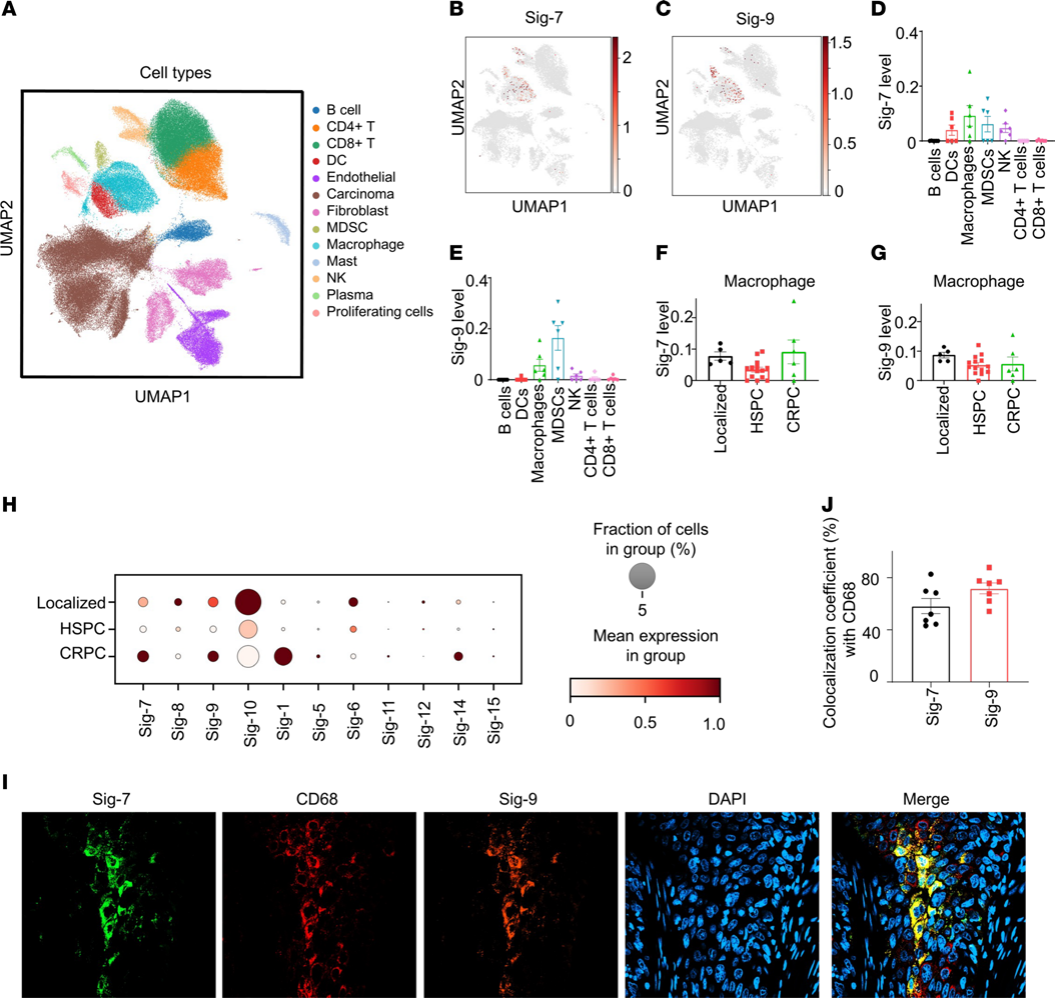
Siglec-7 and Siglec-9 are coexpressed on myeloid cells in human prostate tumors by single-cell RNA-seq. (**A**) UMAP plot showing the distribution of cell types. (**B**) UMAP profiles highlighting Siglec-7 expression, and (**C**) Siglec-9 expression in immune cells of CRPC tumor tissues (*n* = 6). (**D**) Siglec-7 is predominantly expressed in dendritic cells (DCs), macrophages, myeloid-derived suppressor cells (MDSCs), and natural killer (NK) cells in CRPC. (**E**) Siglec-9 is primarily expressed in macrophages and MDSCs in CRPC specimens. (**F**) Siglec-7 and (**G**) Siglec-9 are expressed in macrophages in human tumor tissues from patients with localized PCa (*n* = 5), metastatic HSPC (*n* = 14), and metastatic CRPC (*n* = 6). (**H**) Dot plot illustrates fractional profiles of Siglec expression in immune cells across localized PCa (*n* = 5), metastatic HSPC (*n* = 14), and metastatic CRPC tumor tissues (*n* = 6). (**I**) Confocal microscopy images of a PCa bone metastasis showing the coexpression of Siglec-7 and Siglec-9 on macrophages, observed at 40 × magnification. (**J**) Colocalization coefficient of Siglec-7 or Siglec-9 with the macrophage marker CD68 (*n* = 7).

To elucidate the Siglec profile of PCas, we analyzed 3 different stages of PCa, including 5 primary tumors (localized), 14 metastatic hormone-sensitive tumors and 6 metastatic castration-resistant tumors by single-cell RNA-Seq (scRNA-seq). The cells were annotated (Figure 2A) using published gene signatures (39, 40). Uniform manifold approximation and projection (UMAP) visualization showed that Siglec-7 and -9 are exclusively expressed on immune cells (Figure 2, B and C), mainly on myeloid cells, including macrophages,and myeloid-derived suppressor cells (MDSCs), and sparsely expressed on CD4^+^ T and CD8^+^ T cells (Figure 2, B–E and Supplemental Figure 4) in castration-resistant PCa (CRPC) tumor tissues. Siglec-7 was also highly expressed on DCs and NK cells (Figure 2D). Furthermore, Siglec-7 and Siglec-9 were observed in all stages of PCa, including localized, hormone-sensitive PCa (HSPC) and CRPC (Figure 2, F–H and Supplemental Figures 5 and 6). Additionally, Siglec-10 was highly expressed in macrophages, MDSCs, and B cells across all stages of PCa (Figure 2H and Supplemental Figure 4–6). To validate the expression of Siglec-7 and Siglec-9 on immune cells in PCa, we performed immunofluorescence and confocal microscopy on PCa metastases in bone tissues and found Siglec-7 and Siglec-9 expression on macrophages, with a colocalization coefficient of approximately 65% with CD68 (Figure 2, I and J).

### Sialylated glycans are detected in PCa cells.

**Figure 3:**
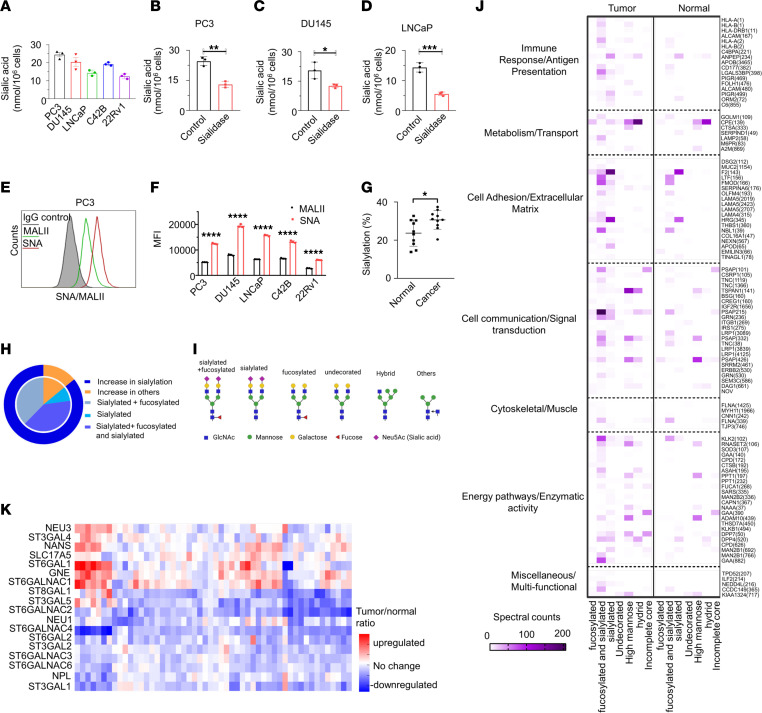
Sialic acid is expressed on the surface of PCa cells. (**A**) surface sialic acid is detected in tested PCa cell lines. Sialidase treatment reduces the surface sialic acid levels in (**B**) PC3, (**C**) DU145, (**D**) LNCaP. (**E**) The expression levels of α2,6-linked and α2,3-linked sialic acids in PC3 cells were analyzed by flow cytometry using Sambucus nigra agglutinin (SNA) and Maackia amurensis agglutinin II (MALII) lectins, respectively. (**F**) Quantification was assessed using MFI for SNA and MALII staining on PCa cells. (**G**) Sialylated spectral counts versus total spectral counts of PCa tumor tissues and adjacent normal tissues by mass spectrometry. (**H**) Summary of glycosylation changes by glycosite in PCa compared with normal prostate samples. (**I**) Schematics of glycan structures. The schematic diagram was created with Biorender.com. (**J**) GO term pathway analysis of sialylated proteins in PCa tumor tissues and adjacent normal tissues by mass spectrometry show enrichment in immune response/antigen presentation pathways. Protein names displayed on the right side with the glycosites in parentheses. (**K**) Analysis of transcript data from TCGA-PRAD database of sialyglycan gene expression demonstrates 7 significantly upregulated genes and 12 downregulated genes in cancer samples compared with noncancerous prostate tissues. Data were analyzed by unpaired Student’s *t* test and presented as mean ± SEM. *P ≤ 0.05; **P ≤ 0.01; ***P ≤ 0.001; ***P ≤ 0.0001.

To explore the presence of sialic acids on the surface of PCa cells, we conducted a sialic acid fluorometric assay using an enzyme-coupled reaction in which the oxidation of free sialic acid generates an intermediate that reacts with a probe, resulting in the production of a detectable fluorescent product. Cell surface sialic acids were present in all tested PCa cell lines (Figure 3A). Furthermore, treatment with sialidase, an enzyme that cleaves sialic acids, led to a significant reduction in surface sialic acid levels in all PCa cells (Figure 3, B–D and Supplemental Figure 7, A and B).

To determine whether the sialic acids present on the cell surface of the PCa cells were α2,3-linked and α2,6-linked, we utilized Sambucus nigra agglutinin (SNA) to detect α2,6-linked sialic acids and Maackia amurensis agglutinin II (MALII) to detect α2,3-linked sialic acids by flow cytometry. All PCa cells stained with both SNA and MALII lectins had higher expression levels of SNA compared with MALII (Figure 3, E and F and Supplemental Figure 7, C–F). This suggests that PCa cells are decorated with both α2,3-linked and α2,6-linked sialic acids, with the surface sialic acids primarily consisting of α2,6-linked sialic acids.

We investigated the sialylation status of proteins in PCa tumor tissues and their adjacent normal tissues. Intact glycoproteomics analysis demonstrated that cancer tissues displayed significantly more sialylated spectral counts (approximately 30%) compared with adjacent normal prostatic tissues (approximately 20%) (Figure 3G). Remarkably, more than 85% of the differential glycosylation between PCa and normal prostate were due to increases in sialylation, including complex sialylated, complex fucosylated and sialylated, or both (Figure 3, H and I). Gene Ontology (GO) term pathway analysis of these glycosylated proteins, including sialylated proteins, demonstrated enrichment in pathways associated with immune response/antigen presentation, metabolism, cell adhesion and communication, and others (Figure 3J). Analysis of transcript expression in sialoglycan biosynthetic genes within tumor tissues from patients with PCa reveals upregulation in 7 genes and a corresponding downregulation in 12 genes, as indicated by the tumor-to-normal tissue ratio (Figure 3K). Across the samples, α-2,3- and α-2,6-sialyltransferase showed substantial modulation across PCa tissues compared with adjacent normal tissues (Figure 3K). K-means clustering based on the correlation between expression of sialoglycan biosynthetic genes and 497 immune genes revealed that the sialoglycan genes could be grouped into 3 distinct gene sets (Supplemental Figure 8). Notably, there is a distinct positive correlation with immune-regulated pathways in gene set 1 including ST3GAL1, and ST6GALNAC4, which are positively correlated with immune system processes, specifically highlighting associations with lymphocytes, leukocytes, and T cell activation (Supplemental Figure 8).

### Siglec-7 and -9 bind to ligands on the surface of PCa cells.

**Figure 4:**
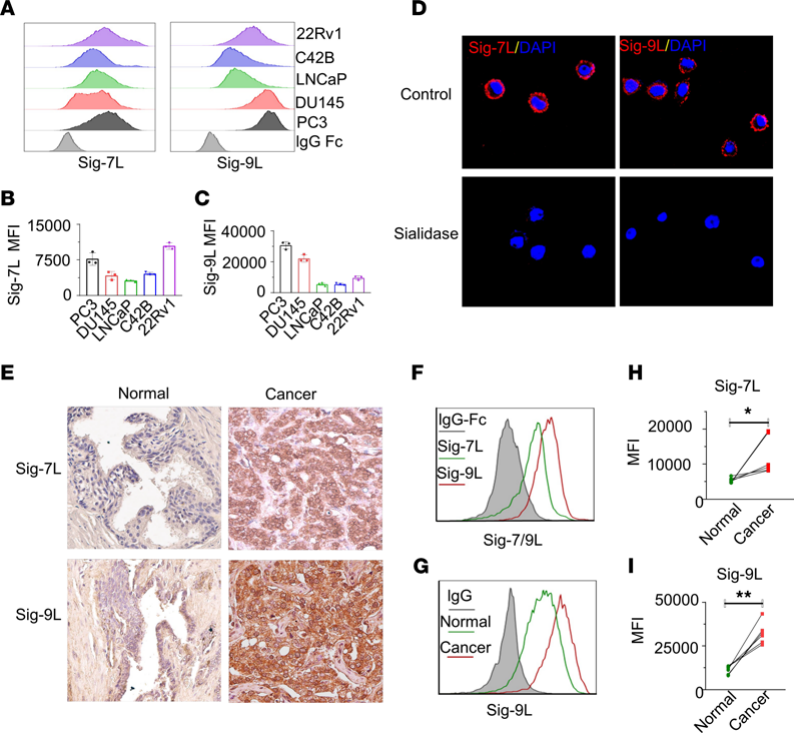
Siglec-7 ligand and Siglec-9 ligand are expressed in PCa cells. (**A**) The expression levels of Siglec-7 ligand (Siglec-7L) and Siglec-9L, and (**B** and **C**) their corresponding quantification by MFI by flow cytometry. (**D**) Confocal microscopy analysis of PC3 PCa cells showing cell surface–associated expression patterns of Siglec-7 and Siglec-9. Treatment with sialidase abolishes the binding of Siglec-7-Fc and Siglec-9-Fc proteins visualized by confocal microscopy, indicating dependence on sialic acid. (**E**) Representative IHC analysis of Siglec-7 and Siglec-9 ligand expression in PCa tumor tissues. High expression levels of Siglec-7 and Siglec-9 ligands are observed in tumor tissues from patients with PCa, while normal tissue samples show either absence or low expression levels.Magnification, × 40. (**F**) Representative Siglec-7 and Siglec-9L expression profile on tumor cells derived from patient tumor tissues by flow cytometry, (**G**) Expression of Siglec-9L expression profile in all cells from tumor tissue, and adjacent normal tissues (*n* = 6). Quantification of (**H**) Siglec-7L and (**I**) Siglec-9L in samples from patients with PCa, suggesting significant higher expression levels of Siglec-7L and Siglec-9L in tumor tissues compared with adjacent normal tissues. Data were analyzed paired Student’s *t* test and presented as mean ± SEM.; *P ≤ 0.05; **P ≤ 0.01.

To determine whether Siglec-7 and Siglec-9 ligands are present on the surface of PCa cells, we employed direct staining using recombinant chimeras of the binding region of Siglec-7 or Siglec-9 molecules fused to human IgG1 Fc domains, referred to as Siglec-7-Fc or Siglec-9-Fc. Flow cytometry analysis revealed high level binding of Siglec-7-Fc to PC3 cells, while 22Rv1 and DU145 cells exhibited moderate Siglec-7-Fc binding levels (Figure 4, A and B). Similarly, a high level of binding of Siglec-9-Fc was observed in PC3 and DU145 cells, whereas 22Rv1 cells displayed moderate Siglec-9 binding (Figure 4, A and C). Conversely, LNCaP and C42B cells showed lower levels of binding of both Siglec-7-Fc and Siglec-9-Fc binding proteins (Figure 4, A and B). In general, all PCa cells exhibited a higher level binding of Siglec-9-Fc compared with Siglec-7-Fc binding (Figure 4, B and C). Removal of sialic acids from the PCa cell surface by treatment with sialidase resulted in a significant reduction in the binding of Siglec-7-Fc and Siglec-9-Fc, as determined by flow cytometry (Supplemental Figure 9). Confocal fluorescence microscopy revealed cell surface binding patterns of Siglec-7-Fc and Siglec-9-Fc on PC3 cells that was abolished by treatment with sialidase, confirming the dependence of the staining on sialic acid (Figure 4D). Furthermore, cell surface localization of Siglec-9 ligands was observed in DU145 and LNCaP cells (Supplemental Figure 10). These results demonstrated that Siglec-7 and Siglec-9 bind to sialic acid ligands on the surface of PCa cells.

Siglec-7-Fc and Siglec-9-Fc binding was also tested directly in human PCa tissues. IHC analysis using the recombinant human Siglec-7 and Siglec-9 Fc chimera demonstrated binding localized to the epithelial cells in PCa tissues, while noncancerous tissues showed either absent or low-level binding of Siglec-7-Fc and Siglec-9-Fc (Figure 4E). We further analyzed Siglec-7 and -9 ligand expression in PCa patient-derived tumor tissues. After digestion of the tissues into single cells by collagenase D, flow cytometry demonstrated that expression levels of Siglec-7 and Siglec-9 ligands were significantly higher in tumor tissues compared with adjacent normal tissues (Figure 4, F–I). Notably, binding of Siglec-9-Fc to PCa tissues was found to be particularly elevated in PCa tissues, consistent with that observed in cultured PCa cell lines. Therefore, in both cultured PCa cells and human tissues, sialic acid ligands that bind to Siglec-7 and -9 are expressed on the cell surface.

### Siglec-7 and -9 blockade inhibits PCa tumor growth in a humanized mouse model.

**Figure 5:**
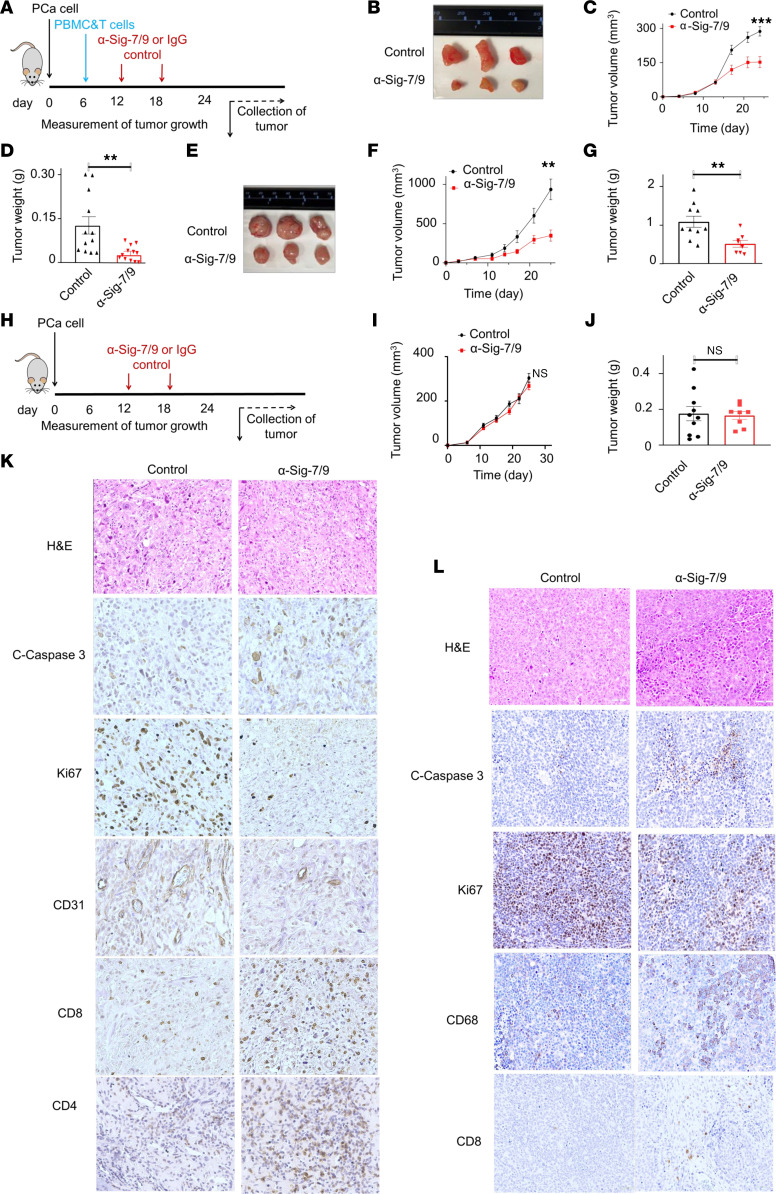
Siglec-7/9 receptor blockade restricts PCa tumor growth in humanized mouse models. (**A**) Schematic diagram depicting the implantation of PCa cells into NSG mice, followed by injection of PBMC and CD8^+^ T cell mixture and subsequent administration of anti-Siglec-7 and anti-Siglec-9 mAbs or IgG antibodies on specific days. (**B**) Representative images of PC3 tumors upon treatment with anti-Siglec-7/9 antibodies (*n* = 12) compared with IgG control (*n* = 12). (**C**) PC3 tumor growth curve and corresponding (**D**) tumor weight. (**E**) Representative images of 22Rv1 tumors upon treatment with anti-Siglec-7/9 antibodies (*n* = 8) compared with IgG control (*n* = 10). (**F**) 22Rv1 tumor growth curve, and corresponding (**G**) tumor weight. (**H**) Schematic diagram depicting control experiment without PBMC and CD8^+^ T cell injection. Control experiment no difference in (**I**) PC3 tumor growth and (**J**) tumor weight between anti-Siglec-7/9 antibody (*n* = 8) and IgG1 isotype control treatment (*n* = 10). (**K**) IHC staining of PC3 tumors treated with anti-Siglec-7/9 antibodies showing increased apoptosis (cleaved caspase 3), decreased proliferation (Ki67), decreased vascularization (CD31), and increased immune cell infiltration, including CD4^+^ and CD8^+^ T cells, compared with IgG1 control. Magnification, × 40. (**L**) IHC staining of 22Rv1 tumors treated with anti-Siglec-7/9 antibodies showing increased apoptosis (cleaved caspase 3), decreased proliferation (Ki67), decreased vascularization (CD31), increased macrophage (CD68) infiltration, and enhanced CD8^+^ T cell numbers compared with IgG1 control. Data were analyzed by unpaired Student’s *t* test and presented as mean ± SEM. **P ≤ 0.01; ***P ≤ 0.001.

To evaluate the role of Siglec-7 and -9 ligands in vivo, we tested whether blocking anti-Siglec-7 and anti-Siglec-9 monoclonal antibodies (mAbs) could control the growth of PCa xenografts. We implanted PC3 cells into immune-deficient NSG mice and allowed tumors to become established for 1 week. Mice were then injected intraperitoneally with human PBMC, enriched with activated human CD8^+^ T cells. On days 13 and 19, the mice were administered anti-Siglec-7 and anti-Siglec-9 blocking mAbs or IgG isotype control via intraperitoneal injection (Figure 5A). By 25 days, PC3 tumor volumes were significantly smaller in the anti-Siglec7/9 mAb treated group compared with the IgG controls (Figure 5, B and C). Tumor weight also was significantly lower in the anti-Siglec7/9 mAb treated group compared with IgG controls at the end of the treatment period (Figure 5D). Similar growth inhibition was observed in AR-expressing 22Rv1 cell line xenografts in anti-Siglec-7/9–treated mice compared with IgG isotype controls (Figure 5, E–G). When we did not inject mice with PBMC and CD8^+^ T cell administration, no significant difference in PC3 tumor growth was observed between animals treated with anti-Siglec-7 and -9 mAb and IgG1 isotype treated controls (Figure 5, H–L), demonstrating that growth suppression was dependent on activity of the human immune cells. IHC staining of PC3 tumor xenografts treated with anti-Siglec-7/9 mAbs demonstrated higher levels of apoptosis (cleaved caspase 3), decreased proliferation (Ki67), vascularization (CD31), and increased immune cell infiltrates, including CD4^+^ and CD8^+^ T cells compared with IgG controls (Figure 5K). Similarly, in the 22Rv1 xenografts, increased apoptosis and decreased proliferation and vascularization were observed in anti-Siglec-7/9 mAb-treated animals compared with isotype controls (Figure 5L). Interestingly, the anti-Siglec-7/9 mAb 22Rv1 tumors showed increased macrophage (CD68) infiltration compared with the IgG control, with fewer T cell infiltrates (Figure 5L). Taken together, these results demonstrate that blocking Siglec-7/9 signaling increases immune responses in PCa.

### CD59 is a candidate Siglec-9 ligand in PCa.

**Figure 6:**
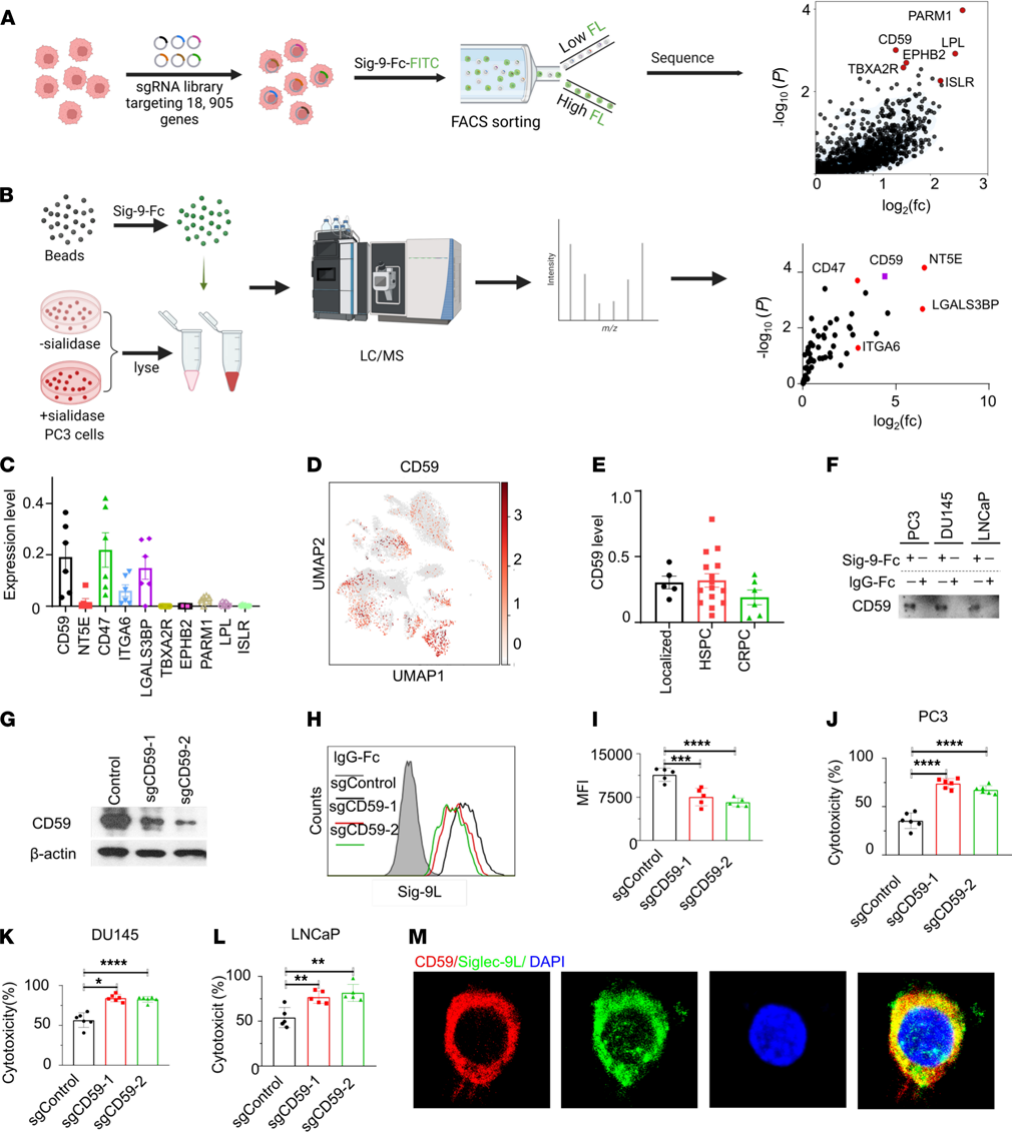
CD59 is a Siglec-9 ligand in PCa. (**A**) Experimental setup and CRISPRi screen results for identifying Siglec-9 ligands using CRISPR screen. PC3 cells expressing dCas9 were infected with a genome-wide CRISPRi-v2 library, and cells with reduced binding to recombinant Siglec-9 Fc proteins were sorted for deep sequencing (*n* = 3). The schematic diagram was created with Biorender.com. (**B**) Identification of sialic acid–dependent Siglec-9 ligands through LC-MS/MS pull down analysis by Siglec-9 Fc chimera protein (*n* = 3). The schematic diagram was created with Biorender.com. (**C**) Analysis of transcript levels of Siglec-9 ligands by sc-RNA sequencing. CD59 and candidate ligands identified by CRISPRi and MS expressed in metastatic epithelial cells from patients with CRPC. (**D**) UMAP profile shows the distribution of CD59 in different cell types in human metastatic CRPC tumor tissues, refer to Figure 2A for cell type distribution. (**E**) CD59 is expressed in cancer epithelial cells from patients with localized, HSPC, and CRPC. (**F**) Western blotting validation of CD59 as a Siglec-9 ligand using recombinant Siglec-9 Fc protein blotting pull-down blotting. (**G**) Western blot analysis of CD59-KO PC3 cells using the CRISPR/Cas9 system. (**H**) Flow cytometry analysis and (**I**) corresponding quantification demonstrating reduced binding capacity of Siglec-9 Fc in CD59 knockout PC3 cells compared with control cells. Gray, IgG Fc; black, control cells; red, sgCD59-T1; green, sgCD59-T2. (**J**) Enhanced cytotoxicity of CD8^+^ T cells were observed in CD59-KO PC3 cells, (**K**) DU145 cells, and (**L**) LNCaP cells. (**M**) Confocal microscopy showing substantial colocalization between Siglec-9 ligands and CD59 on PC3 cells. Data were analyzed by 1-way ANOVA with post hoc Tukey’s test and presented as mean ± SEM.*P ≤ 0.05; **P ≤ 0.01; ***P ≤ 0.001; ***P ≤ 0.0001.

Since PCa cells exhibit higher binding of Siglec-9 compared with Siglec-7, we sought to identify Siglec-9-binding glycoproteins as candidate ligands. We first performed a CRISPRi screen to in which we FACS sorted cells in which targeted knockdown of proteins substantially diminished Siglec-9-Fc binding. Specifically, we transduced PC3 cells expressing dCas9 with a genome-wide human CRISPRi-v2 library containing 209,070 single guide RNAs (sgRNAs) that target 18,905 genes (10 sgRNAs/gene) (Figure 6A) (41). Genomic DNA was isolated from the sorted PC3 cells that exhibited reduced binding to Siglec-9 Fc proteins obtained by FACS for subsequent deep sequencing. Notably, CD59, a sialylated glycoprotein, was identified as one of the top candidate ligands for Siglec-9, along with other top candidates such as PARM1, EPHB2, LPL, ISL, and RTBXA2R (Figure 6A).

To further identify Siglec-9 ligands, we used Siglec-9-Fc coupled with protein G beads to immunoprecipitate Siglec-9 ligands from PC3 cell lysates that were untreated or pretreated with sialidase. Interacting proteins from both sample sets were identified via shotgun proteomics. Proteins that were significantly enriched in the untreated compared with the sialidase-treated samples were identified as sialic acid–dependent ligands for Siglec-9. We identified CD47, CD59, CD73 (also known as NT5E), and LGALS3BP as potential candidate cell surface ligands interacting with Siglec-9 (Figure 6B). Once again, CD59 was one of the top candidate ligands identified by liquid chromatography–tandem mass spectrometry (LC-MS/MS), implicating CD59 as a potential ligand for Siglec-9.

The scRNA-seq analysis revealed that candidate Siglec-9 ligands CD59, CD47, and LGAL3BP were expressed in tumor cells in metastatic tumor tissues derived from patients with CRPC (Figure 6C). UMAP visualization showed that CD59 was expressed in various types of cells including tumor cells in human localized, metastatic HSPC, and CRPC tumor tissues (Figure 6D and Supplemental Figure 11). CD59 is expressed in cancer cells in localized, HSPC, and CRPC tumor tissues (Figure 6E).

To validate CD59 as a ligand for Siglec-9, we performed Western blotting on the Siglec-9-Fc–captured proteins from PC3, DU145, and LNCaP PCa cell lines using anti-CD59 antibodies. As depicted in Figure 6F, CD59 protein band was clearly observed in the protein lysates of PCa cells captured using recombinant Siglec-9 Fc protein, while no band was detected in the isotype IgG Fc pull-down samples. To further validate the binding between Siglec-9 and CD59 on the cell surface, we generated CD59-knockout PC3 cells using the CRISPR/Cas9 system. The CD59 protein level was significantly reduced in sgCD59-transduced cells as detected by Western blot analysis (Figure 6G). Flow cytometry analysis demonstrated a reduced capacity of Siglec-9 Fc binding in the CD59-knockout PC3 cells compared with the control cells (Figure 6, H and I). Moreover, CD8^+^ T cell cytotoxicity expressed with high level of Siglec-7 and Siglec-9 (Supplemental Figure 12) was enhanced following CD59 knockout in PC3 cells (Figure 6J). These findings were also replicated in other PCa cell lines including DU145 and LNCaP cells (Figure 6, K and L), providing further evidence for the role of CD59 as a functional ligand for Siglec-9 in PCa cells. Immunofluorescent staining of PC3 cells with both the Siglec-9-Fc–FITC conjugated antibody and anti-CD59 antibody conjugated with PE confirmed cell surface colocalization of CD59 and Siglec-9 binding (Figure 6M), supporting the notion that CD59 is a potential Siglec-9 ligand in PCa cells.

## Discussion

Although PCa has been classified as an “immune cold” malignancy because of very limited immune cell infiltration (42), it was the first cancer for which immunotherapy was FDA approved after demonstration of improved patient survival with Sipuleucel-T in 2 phase III clinical trials (10, 11). In this therapy, leukapheresed peripheral monocytic cells, including antigen presenting cells, are expanded and exposed to the prostate specific glycoprotein prostatic acid phosphatase (PAP) and reinfused into men with metastatic PCa. The finding that a glycoprotein can elicit an immune-response in PCa suggests a latent ability to activate the immune system against PCa through a glycan-mediated immunosuppressive pathway. We propose that substantial changes in sialylation of cell surface proteins in PCa that interact with Siglec receptors on immune cells is 1 pathway through which glycoproteins suppress immunity in PCa. We demonstrate the enrichment of sialic acids that can serve as ligands for Siglec-7 and Siglec-9 in PCa cells and human tissues. Our findings reveal that Siglec-7 and Siglec-9 are highly expressed in myeloid cells, including macrophages. Blocking of Siglec-7 and Siglec-9 in PCa xenografts decreased cancer growth and was dependent on the presence of immune cells. Finally, we identify CD59 as a candidate ligand for Siglec-9 in PCa, highlighting a potential target for a novel immunotherapy.

A growing body of evidence implicates the sialic acid/Siglec pathway in immunosuppression in human malignancies (20, 24, 26, 28, 43). Siglec ligands have been identified on many cancer cell types and invariably have been shown to suppress the activation of multiple subsets of immune cells in diverse cancer types (44, 45). As we observed in PCa, blocking of Siglec-7, Siglec-9, or both can result in tumor shrinkage in melanoma and ovarian cancer models (24, 46). In addition, Siglec-9 is detected in many human cancers and high Siglec-9 expression is correlated with short progression-free survival (23). For example, Siglec-7 and -9 are highly expressed on myeloid cells in pancreatic ductal adenocarcinoma (PDA) (20). Elevated Siglec-9 is reported in PBMC derived CD8^+^ and tumor infiltrating lymphocytes in patients with non-small cell lung cancer (NSCLC) (34). In PCa, we found that high expression of Siglec-7 and Siglec-9 are associated with poorer progression free survival as well as increased cancer grade, suggesting an immunosuppressive role.

The mechanisms of immunosuppression in cancers via Siglec signaling appear to be diverse and complex. For example, in human leukemia, binding of CD43 to Siglec-7 receptors on NK cells appears to be a critical mechanism of immunosuppression (44). In PCa, our transcriptomic, in vitro and in vivo data implicates myeloid cells, including macrophages and MDSCs, which express high levels of Siglec-7 and Siglec-9 in shaping the PCa immune landscape. In PCa, upregulation of MYC signaling pathways are some of the most common genetic alterations observed, often in parallel with MYC amplifications (47). We have previously reported that modulation of MYC signaling increases cancer cell sialylation through upregulation of sialytransferases, particularly ST6GALNAC4 (48). In lymphocytic leukemia cell lines, these alterations result in changes in tumor infiltration of NK cells, macrophages, CD4^+^ T cells, and CD8^+^ T cells, and increased CD69 expression on both CD4^+^ and CD8^+^ T cells. We observed similar effects following Siglec blockade in our in vivo studies using PCa xenografts and human PBMCs. Additional investigation into the effects of sialic acid/Siglec interactions in PCa will be necessary to elucidate broad immune-modulatory effects of this pathway.

We identify CD59 as a candidate ligand for Siglec-9 in PCa by 2 independent methods, suggesting it could be a therapeutic target in PCa. Our observation that CD59 acts as an immune-suppressive Siglec-9 ligand agrees with previous observations that protein expression of CD59 in human PCa (49) and other solid tumors, including colon cancer (50), breast cancer (51), NLCLC cancer (52), and esophageal squamous cell carcinoma (53) is associated with adverse outcomes. High protein CD59 expression is associated with poor survival outcomes in PCa (49). Whether targeting CD59 would be effective in PCa treatment is uncertain and requires additional investigation. CD59 has several additional functions relevant to immune function, including the complement activation cascade where it binds to C8 and C9 to inhibit the formation of membrane attack complex (MAC) pores, conferring cancer cells resistant to complement-mediated cell lysis (54, 55). In addition, it is possible that there are additional Siglec ligands active in PCa immunosuppression. Both the CRISPRi screen and the pull-down experiments showed several additional candidate ligands that could be functional when CD59 is blocked. Alternatively, these ligands could be active in different contexts, such on specific immune cells or in particular microenvironments. An alternative therapeutic strategy would be to target Siglec receptors directly, using therapies such as with receptor-blocking antibodies used in this study. In addition, we have developed a bifunctional protein with a ligand that binds cell surface proteins on cancer cells to direct a sialidase specifically to the cancer cell surface and demonstrated efficacy in targeted HER2 in breast cancer (56, 57). A similar therapeutic strategy could be applied to PCa by taking advantage of prostate-specific membrane antigen (PSMA) or Prostate stem cell antigen (PSCA) proteins for specific targeting. Our findings highlight the importance of Siglec-7/9 expression and their interactions with sialic acids on Siglec-7/9 ligands in PCa progression and suggest potential therapeutic targets including CD59/Siglec-9 for immune-based therapeutic interventions.

## Methods

The materials, including antibodies and agents, are listed in Table 1 and Supplemental Table 1.

### Sex as a biological variable.

Since PCa is a male-specific disease, only prostate tumor tissues from men and male mice were used in this study.

### Patient samples.

PCa tissue samples were obtained from the Stanford Tissue Bank and UCSF Cancer Immunotherapy Program Biobank under an IRB approved protocol and stored at –80°C prior to analysis. Blood samples from donors who did not have a history of immunologic disorder or had been treated with immunomodulatory drugs within 6 months were obtained from the Stanford Blood Bank. All patient samples were deidentified for the study.

### Cell lines and culture conditions.

PC3, DU145, LNCaP, C42B, 22Rv1 cells, and embryonic kidney 293T cells were obtained from ATCC. PC3, LNCaP, C42B, and 22Rv1 cells were cultured in RPMI-1640 medium (Invitrogen) supplemented with 10% FBS and 100 U/mL penicillin/streptomycin (Invitrogen). 293T and DU145 cells were cultured in DMEM (Invitrogen) containing 10% FBS and penicillin/streptomycin (100 U/mL). All cells were maintained in a humidified 5% CO_2_ incubator at 37°C and were regularly monitored for mycoplasma contamination and morphological changes.

### PBMC isolation.

Peripheral blood mononuclear cells (PBMCs) were separated from whole blood using Lymphocyte Separation Medium (Corning). The whole blood was diluted in PBS (1:3) and layered onto 15 mL lymphocyte separation medium, and centrifuged at 400*g* for 20 minutes at low speed. The PBMCs were collected from the interface layer. The PBMCs were washed with PBS (1×), and collected by centrifuge at 300*g* for 10 minutes.

### Tumor digestion.

PCa patient tumors were finely minced and immersed in a digestion medium comprising RPMI with 2% FBS, collagenase IV (1 mg/mL), and DNase I (100 μg/mL). After incubation at 37°C with intermittent shaking, the digested tissues were processed through a 100 μm cell strainer to yield a single-cell suspension. The cells were washed using FACS buffer containing 2% fetal calf serum and 2 mM EDTA in PBS and centrifuged at 300*g* at 4°C.

### Flow cytometry.

Siglec-7/9 ligand expression was detected using recombinant chimeras of the Siglec-7 or Siglec-9 binding region fused to human IgG Fc domains (Siglec7/9-Fc). Specifically, Alexa Fluor 488 anti-human IgG and Siglec-7-Fc or Siglec-9-Fc chimeric protein were diluted to a final concentration of 1 μg/mL in PBS with 0.5% BSA) and incubated on ice for 1 hour to form Siglec-7-Fc/anti-human lgG- Alexa Fluor 488 or Siglec-9-Fc/anti-human lgG-Alexa Fluor 488 conjugates. PC3, DU145, LNCaP, C42B, and 22Rv1 cells were detached using accutase (Innovative Cell Technologies). Cells were washed, pelleted, and resuspended in the above Siglec-7 or Siglec-9-Fc/anti-human lgG-Alexa Fluor 488 precomplex solution at a density of 10 × 10^6^ cells/mL. After incubation on ice for 30 minutes, the cells were pelleted by centrifugation at 300*g* for 5 minutes and washed twice and resuspended in PBS containing 2% FBS and 1mM EDTA. Cells treated with sialidase for 30 minutes in serum free medium were used as a negative control. Cell surface staining with FITC-conjugated SNA and MAA/MALII were performed similarly except that the cells were stained in SNA-FITC(10 μg/mL) or MALII (20 μg/mL) on ice for 1 hour. The cells were washed and resuspended in PBS containing 2% FBS and 1 mM EDTA.

PC3 and DU145 cell sorting was performed using a BD FACSAria II (BD Bioscience) to isolate specific cell populations excluding doublets, cell debris, and dead cells. Fluorescence-minus-1 (FMO) samples were utilized to define the gate strategy for positive cells.

### Sialic acid assay.

The sialic acid levels in PCa cells were quantified using a sialic acid assay kit (ab83375, abcam) according to manufacturer’s instructions. Briefly, PC3, DU145, LNCaP, C42B, and 22Rv1 cells were detached using cell scrapers, the cells (50,000 cells/well) were transferred into 96-well plates and a working solution (50 μL), composed of sialic acid converting enzyme, sialic acid development mix, and sialic acid probe, was added into plates. After incubating for 30 minutes at room temperature, the fluorescence signal intensity was measured at 535/537 nm.

### Confocal microscopy.

PC3, DU145, and LNCaP cells were plated onto μ-Slide 8 well glass bottom chamber slides (Ibidi USA) and cultured overnight. The cells were fixed in 4% formaldehyde solution in PBS and treated with buffer or sialidase for 30 minutes at room temperature. After blocking nonspecific binding using anti-human Fc blocking agent in PBS containing 5% goat serum for 1 hour, cells were incubated with Siglec-7-Fc or Siglec-9-Fc/anti-human IgG R-Phycoerythrin conjugates and imaged using confocal microscopy (ZEISS 880 or ZEISS 980). Colocalization of CD59 with Siglec-9 ligands in PC3 cells was determined by costaining using Siglec-9-Fc-FITC and anti-CD59-PE antibodies.

### Plasmid constructs.

The dCas9, and hCRISPRi-v2 libraries originally generated by Dr. Irving Weissman’s group were procured from Addgene (41). The GPF plasmid was obtained from Sino Biologicals. The sgRNAs targeting CD59 were obtained from ABM Goods Inc.

### Lentiviral production and transduction.

All lentiviruses were generated using a third-generation system, as described previously (58). Briefly, 293T cells were cultured in the presence of cloroquine diphosphate at a final concentration of 50 μM for 5 hours. Lentiviral constructs (17 μg) were mixed with pMDL (11 μg), VSV-G (6 μg), and pREV (4 μg) in HEPES buffered saline (Alfa Aesar) with CaCl_2_ (Sigma-Aldrich) at a final concentration of 0.5M, and the resulting DNA-calcium phosphate coprecipitate was added onto 293T cells. After incubating overnight, medium was removed and replenished. Two days later, medium containing lentiviruses was collected and concentrated using a PEG-it kit (LV810A-1, System Biosciences).

PCa cells were transduced by lentiviral particles in medium containing polybrene at a concentration of 8 μg/mL for 3 days. For CRISPRi-v2 library transduction, PC3 cells were first transduced with dCas9 lentiviral particles. Genome wide human CRISPRi-v2 libraries containing with 209,070 sgRNAs that target 18,905 genes (10 sgRNAs per gene) were then transduced into PC3 cells expressing dCas9. This workflow enabled library coverage of 1,000× with 2 × 10^8^ cells.

### FACS sorting of cells with low Siglec-9-Fc binding.

The CRISPRi-v2 library transduced PC3 cells (2 × 10^8^) were washed, centrifuged, and stained with Fixable Viability Dye eFluor 450 (65-0863-14, eBioscience), followed by staining with precomplexed Siglec-9-Fc/anti-human lgG-Alexa Fluor 488 for 30 minutes on ice. Stained cells were resuspended in FACS buffer consisting of 1 mM EDTA and 2% FBS in PBS and filtered through a cell strainer (100 μM). Cells that passed through the strainer were sorted using a BD FACS Aria II with an event rate of 2,000–4,000 cells/second. Live cells with low fluorescence (defined as approximately 10% of the parent Siglec-9-Fc-staining were collected.

### Library amplification, sequencing, and data processing.

The genomic DNA (gDNA) was isolated from sorted cells expressing low levels of Siglec-9 ligands and unsorted control samples using a NucleoSpin Blood XL kit (740950.50, Macherey-Nagel) following the manufacturer’s instructions. Specifically, the harvested unsorted cells or sorted cells were centrifuged at 400*g* for 5 minutes and followed by resuspension in PBS at 10 mL/100 million cells. Proteinase K (0.5 mL/100 million cells) and BQ1 buffer (10 mL/100 million cells) were added into resuspended cells. The cells were incubated at 56°C for 1 hour and were then cooled to room temperature before adding 100% ethanol buffer (10 mL/100 million cells). DNA was extracted from the lysates using a NucleoSpin Blood column centrifuged at 3,000*g* for 3 minutes. The DNA was washed with BQ2 buffer (10 mL) twice, and the silica membranes were dried by centrifuging at 3,000*g* for 10 minutes. The DNA was eluted with 70°C BE buffer and centrifuged at 3,000*g* for 5 minutes (1 mL/column).

The gDNA concentrations were determined using a Nanodrop 2000 instrument (Thermo Fisher Scientific), and gDNAs were amplified with TruSeq indices by PCR using NEBNext Ultra II Q5 MasterMix (New England Biolabs, no. M0544L) for 22 cycles at 98°C for 10 s and 65°C for 75 s.

Double SPRI purification using SPRIselect Reagent (Beckman and Coulter) was performed to remove the gDNA and primer background from the PCR by selecting 274 bp fragments (59). Specifically, 0.65 × SPRI beads were added into the PCR reaction solution to remove the fragments over 300 bp. The suspension was collected, and 1× SPRI beads were added to select fragments over 150 bp. The beads were washed with 80% ethanol, air dried for 10 minutes, and eluted with EB buffer (20 μL).

The purified PCR products were sequenced with 10% Phix Spike-in on an Illumina Hiseq4000 by Novogene (41). The FASTQ sequences were demultiplexed and aligned to the reference genome using MAGeCK (60). The sgRNA sequences in samples with low Siglec-9-Fc-FITC staining were compared with unsorted control samples to identify enriched sequences. Genes encoding cell surface (GO:0009986) and glycoprotein were used to select Siglec-9 ligands.

### Siglec-9-Fc pulldown and mass spectrometry.

PC3 cells were harvested using a cell scraper and centrifuged at 300*g* for 5 minutes. The cells were washed with PBS (1 ×) and resuspended in lysis buffer (0.1% NP-40 in PBS with 1 × Halt protease inhibitor, Thermo Fisher Scientific) at a concentration of 100 mg/mL on ice, followed by sonication using a probe sonicator. The insoluble fraction was pelleted at 20,000*g* and supernatants were collected to quantify the protein concentration by Pierce BCA Protein Assay Kit (Thermo Fisher Scientific) according to manufacturer’s instructions. The samples were diluted in lysis buffer at 1 mg/mL. The samples were incubated with or without 100 nM *V*. *cholerae* *sialidase* for 3 hours at 37°C. The Siglec-9-Fc protein (5 μg) was complexed with Protein G beads (50 μL) in PBS (250 μL) for 1 hour at room temperature. The Siglec-9-Fc functionalized beads were washed with PBS (1×). The sialidase-treated or untreated lysates were added into Siglec-9-Fc functionalized beads and incubated at 4°C overnight with continuous rotation. The next day, the beads were washed with lysis buffer (2 ×) and 50 mM (triethyl)ammonium bicarbonate (3 ×). The proteins were eluted by boiling the beads at 95°C in 50 μL (triethyl)ammonium bicarbonate with 0.05% Rapigest. DTT was added to the eluate, which was then incubated at 60°C for 30 minutes, followed by alkylation with 10 mM iodoacetamide at room temperature for 30 minutes, digestion by trypsin (1 μg) in 50 mM (triethyl)ammonium bicarbonate buffer at 37°C overnight, and acidification by formic acid at a final concentration of 2% at 37°C for 30 minutes. The samples were dried in a speedvac overnight. The samples were cleaned using a Strata-X column, dried using a speedvac, and resuspended in 10 μL 0.2% formic acid.

Peptides were separated over a 25 cm Aurora Series Gen2 reverse-phase LC column (75 μM inner diameter packed with 1.6 μM FSC C18 particles, Ion Opticks). The mobile phases (A: water with 0.2% formic acid and B: acetonitrile with 0.2% formic acid) were driven and controlled by a Dionex Ultimate 3000 RPLC nano system (Thermo Fisher Scientific). An integrated loading pump was used to load peptides onto a trap column (Acclaim PepMap 100 C18, 5 μm particles, 20 mm length, Thermo Fisher Scientific) at 5 μL/minute, which was put in line with the analytical column 5 minutes into the gradient. The gradient was held at 0% B for the first 6 minutes of the analysis, followed by an increase from 0% to 5% B from 6 to 6.5 minutes, an increase from 5 to 22% B from 6.5 to 66.5 minutes, an increase from 22% to 90% from 66.5 to 71 minutes, isocratic flow at 90% B from 71 to 75 minutes, and reequilibration at 0% B for 15 minutes for a total analysis time of 90 minutes. Eluted peptides were analyzed on an Orbitrap Fusion Tribrid MS system (Thermo Fisher Scientific). Precursors were ionized with a spray voltage held at +2.2 kV relative to ground, the column was held at a constant temperature of 40°C using a sonation column oven, and the inlet capillary temperature was held at 275°C. Survey scans of peptide precursors were collected in the Orbitrap from 350–1350 Th with an AGC target of 1,000,000, a maximum injection time of 50 ms, RF lens at 60%, and a resolution of 60,000 at 200 m/z. Monoisotopic precursor selection was enabled for peptide isotopic distributions, and precursors of z = 2–5 were selected for data-dependent MS/MS scans for 2 seconds of cycle time. Dynamic exclusion was set to exclude precursors after being selected once for an exclusion time of 30 seconds with a ± 10 ppm window set around the precursor monoisotope. An isolation window of 1 Th was used to select precursor ions with the quadrupole, and precursors were fragmented using a normalized HCD collision energy of 30. MS/MS scans were collected with an AGC target of 100,000 ions, with a maximum accumulation time of 54 ms and an Orbitrap resolution of 30,000 at 200 m/z. The same method was used for both untreated and sialidase-treated samples. Raw data were processed with MaxQuant version 1.6.2.10 (61) and tandem mass spectra were searched with the Andromeda search algorithm (62). 20 ppm, 4.5 ppm, and 20 ppm were used for first search MS1 tolerance, main search MS1 tolerance, and MS2 product ion tolerance, respectively. Oxidized methionine and deamidated asparagine were set as variable modifications, and carbamidomethylation of cysteine was set as a fixed modification. Cleavage specificity was set to Trypsin/P with 2 missed cleavages allowed. Peptide spectral matches (PSMs) were made against a human protein database (reviewed entries only, 20,416 entries total) downloaded from Uniprot. Peptides were filtered to a 1% FDR using a target-decoy approach (63), and a 1% protein FDR was applied. Proteins were quantified and normalized using MaxLFQ, and the match between runs feature was enabled. Label free intensity values were log_2_ transformed using Perseus version 1.6.2.2. (64), protein groups with signal in all 3 replicates of at least 1 condition were kept, and missing values were imputed using default settings of a distribution downshifted by 1.8 with a width of 0.3 σ.

### Siglec-9 Fc pulldowns and Western blotting.

PC3, DU145, and LNCaP cells were lysed in IP buffer (Thermo Fisher Scientific). Siglec-9 Fc or human-lgG1-Fc (5 μg/sample) were conjugated to Protein G beads (50 μL/sample) at 4°C for 1 hour. The conjugated beads were added to the cell lysates and incubated at 4°C for 3 hours with gentle shaking. The beads were collected and washed with cell lysis buffer. The bound proteins were eluted by boiling beads in 1 × Laemmli Sample Buffer (Bio-Rad) for 10 minutes prior to loading onto a 4%–12% TrisGlycine Gel. Western blotting was performed with an anti-CD59 primary antibody (Sigma-Aldrich) at a 1:1,000 dilution in TBS-T overnight at 4°C. The membrane was incubated with secondary antibody (anti-Rabbit HRP) for 1 hour at room temperature. Enhanced chemiluminescence (ECL) was used to develop the membrane for imaging.

### In vivo analysis of Siglec-7/9 function.

NSG (NOD/LtSz-SCID IL-2Rγnull) mice were procured from the Jackson Laboratory and housed at the Veterinary Service Center of Stanford University. PC3 and 22Rv1 (1 million cells) were injected subcutaneously into the flank of 8-to-10 week-old male mice at day 0. CD8^+^ T cells (2 million/mouse) activated by anti-CD3/CD28 beads for 4 days were coadministered with PBMCs (10 million/mouse) on day 7 by intraperitoneal injection. The mice engrafted with tumor cells were randomly assigned into 2 groups. The anti-Siglec-7/9 or isotype control (100 μg/mouse) were injected intraperitoneally into mice at day 13 and day 19. Tumor size was measured every 3 days using a caliper, and tumor volume was calculated as V = 1/2 L × W^2^. At the end of the experiment, tumor tissues were collected for further analysis.

### Staining for Siglec-7 and Siglec-9 ligands on tumor tissue.

Tumors were fixed in formalin and sectioned at a thickness of 5 μM. The tissue slides were incubated in accutase for 10 minutes at 37^o^C for antigen retrieval. The slides were then blocked in a series of blocking buffers: 5% goat serum and 5% BSA in PBS (1 hour), avidin blocking buffer (15 minutes), biotin blocking buffer (15 minutes), and anti-human Fc blocker in PBS buffer containing 0.5% BSA (1 hour) (all from Thermo Fisher Scientific). Next, the slides were incubated with Siglec-7-Fc or Siglec-9-Fc chimera protein at 4°C overnight, followed by endogenous peroxidase and alkaline phosphatase blocking for 10 minutes using dual Endogenous Enzyme Block solution (Dako). Finally, the tissue slides were washed and incubated with anti-human lgG HRP at room temperature for 1 hour. All other steps are the same as above IHC staining.

### Transcriptome data analysis.

TCGA gene expression data from primary tumors and matched adjacent normal tissues of 52 patients diagnosed with PRAD were obtained from the Broad Institute. The raw data, comprising 497 primary tumor samples, were processed by normalizing to transcripts per million (tpm) and applying log transformation (log_10_(1 + tpm)) due to the long-tailed gene expression distributions. Subsequently, log fold changes (lfc) for each gene were calculated as the difference between the log-transformed values of the primary tumor and adjacent normal tissue, lfc = log_10_ ((+ tpm cancer) – log_10_((+ tpm normal). A 1-sample *t* test was employed to assess the null hypothesis that the lfc for each gene is 0 (scipy.stats.ttest_1samp). Multiple hypothesis correction was performed using the Benjamini-Hochberg procedure (α threshold of 0.1).

Analysis was performed in python (ver. 3.11.7) with scikit-learn (1.3.2), scipy (ver. 1.11.4), and lifelines (ver. 0.28.0). Code is accessible through github (https://github.com/emarti/siglec/; commit ID: 952ffb4098e6c9f0cc7ba0af42d50b49cf1c19c8). GO terms annotations are from the basic version of GO (“go-basic.obo” ver. 1.2, 2023-10-09 release) with human annotations from GOA human (“goa_human.gaf”, 2023-10-10 release).

To obtain clinical information, we retrieved the TCGA gene expression profile (Prostate Adenocarcinoma, PanCancer Atlas) from 494 primary tumors using cBioPortal (https://www.cbioportal.org/). Kaplan-Meier survival analysis was conducted on 488 tumor samples that included clinical survival follow-up data. The patients were grouped into low/high Siglec-7/9 based on the median expression levels. RNA-seq data from TCGA-PRAD were deconvoluted into individual cell types using CIBERSORTx (38). For each reference dataset, a signature matrix LM22 containing 547 genes was computed using CIBERSORTx. TCGA-PRAD RNA-seq data were used as an input for deconvolution. CIBERSORTx was operated in “absolute mode” to normalize deconvolution results into percentages.

### LC-MS/MS glycopeptide analysis.

Enriched glycopeptides from PCa and benign adjacent (BA) tissues were analyzed by LC-MS/MS using a Dionex UltiMate 3000 RSLCnano system coupled to a LTQ-Orbitrap Elite mass spectrometer (Thermo Fisher Scientific). A 10 μL aliquot of the glycopeptide-enriched sample was loaded onto a 5 mm Acclaim PepMap 100 C18 trap column (Thermo Fisher Scientific) at a constant flow rate of 5 μL/min for 10 minutes. Glycopeptides were then separated by reversed phase liquid chromatography on a 25 cm analytical column packed in house with Magic C18, 100 Å packing material (Michrom Bioresources). The chromatographic gradient consisted on holding buffer A (0.1% formic acid in water) for 10 minutes (loading step) at 98% and increasing buffer B (0.1% formic acid in ACN) from 2 % to 35% in 90 minutes. A rapid increase of buffer B to 85% in the next 10 minutes with a 5 minute hold before bringing down buffer B to initial conditions of 2% for column equilibration before the next sample injection. Eluting glycopeptides were ionized using a nanospray Flex ion source voltage with the voltage set to 1.5 kV and the heating capillary temperature set to 200°C. The mass spectrometry analysis parameters were set as follows: a full MS scan was performed in the FT mass analyzer from 400 to 1800 m/z with a mass resolution of 30,000, and the MS/MS fragmentation was performed using higher-energy collision-induced dissociation (HCD) on the top 10 most abundant precursor ions in the MS1 scan. Dynamic exclusion was enabled, with a 30 second exclusion duration. The following conditions were used for HCD activation: 35 normalized collision energy, 4 m/z isolation width, 0.40 ms activation time, and a minimal precursor signal threshold of 1,000 counts.

### Single cell RNA-seq analysis.

Tissues were obtained from patients with localized PCa (NCT03821246), metastatic HSPC (NCT03007732), and metastatic CRPC (NCT03248570), and further processed for scRNA-seq by protocols described previously (39, 40). The raw data from 10 × sequencing were processed using the Cell Ranger pipeline (v3, Genome build: GRCh38). The gene expression matrices were subjected to ambient RNAs removal by CellBender (v0.1.0) (65), doublet detection by the package DoubletDetection (10.5281/zenodo.2678041), and then analyzed through the SCANPY pipeline (65) with batch correction by the package Harmony (66). Leiden clustering (default resolution = 1.0), and UMAP plotting were performed for cell clustering. Differential expression analysis identified top-ranked genes upregulated in each individual cluster relative to the combination of all other cells, as determined by the SCANPY function tl.rank_genes_groups. Annotation of each unbiased population was achieved through manual inspection of the top-ranked genes of each cluster. The selected gene expressions on UMAPs were performed by SCANPY function pl.umap. To calculate gene expression at the sample level, the mean expression of cells in each cell cluster from each sample was calculated and then plotted using GraphPad Prism software.

### Statistics.

Statistical analyses were performed using 1-way ANOVA with post hoc Tukey’s test for more than 2 groups. An unpaired or paired Student’s *t* test (2-tailed) was used for comparison of 2 groups, as indicated in the figure legends. Survival analysis was conducted using the Kaplan–Meier (log-rank test). Median expression was used as cutoff between high and low Siglec-7 and -9 expressing groups. Data were presented as mean ± SEM. Statistical significance was defined as *P* under 0.05 was statistically significant.

### Study approval.

The use of human specimens received approval from the Institutional Review Board of Stanford University (IRB no. 59488). The use of animals in the study was following the guidelines and regulations set forth by the Institutional Animal Care and Use Committee (IACUC) of Stanford University (12944).

### Data availability.

CRISPRi screen data are available in the Supporting Data Values file. The scRNA-seq data was deposited into NCBI Gene Expression Omnibus (GEO) with accession number GSE274229. We have used published TCGA datasets, which could be downloaded from Broad Institute and the data with clinical information was obtained from cBioPortal (dataset: “Prostate Adenocarcinoma (TCGA, Firehose Legacy)”). All other data are available in Supporting Data Values supplemental material.

## Author contributions

RMW designed and conducted experiments, analyzed data, drafted the manuscript, and oversaw the project. JCS, ZF, AL, NMR, SMT, AB, RN, and XZ contributed to the conduct of experiments. GEWM, FJGM, and ZF performed bioinformatic analyses of gene expression and proteomic data. HZ, LF, SJP, EGE, CRB, and JDB designed the studies and oversaw the project. XZ, JCS, NMR, SJP, HZ, and JDB revised the manuscript. All authors read and approved the final manuscript.

## Supplementary Material

Supplemental data

Unedited blot and gel images

Supporting data values

## Figures and Tables

**Table 1:**
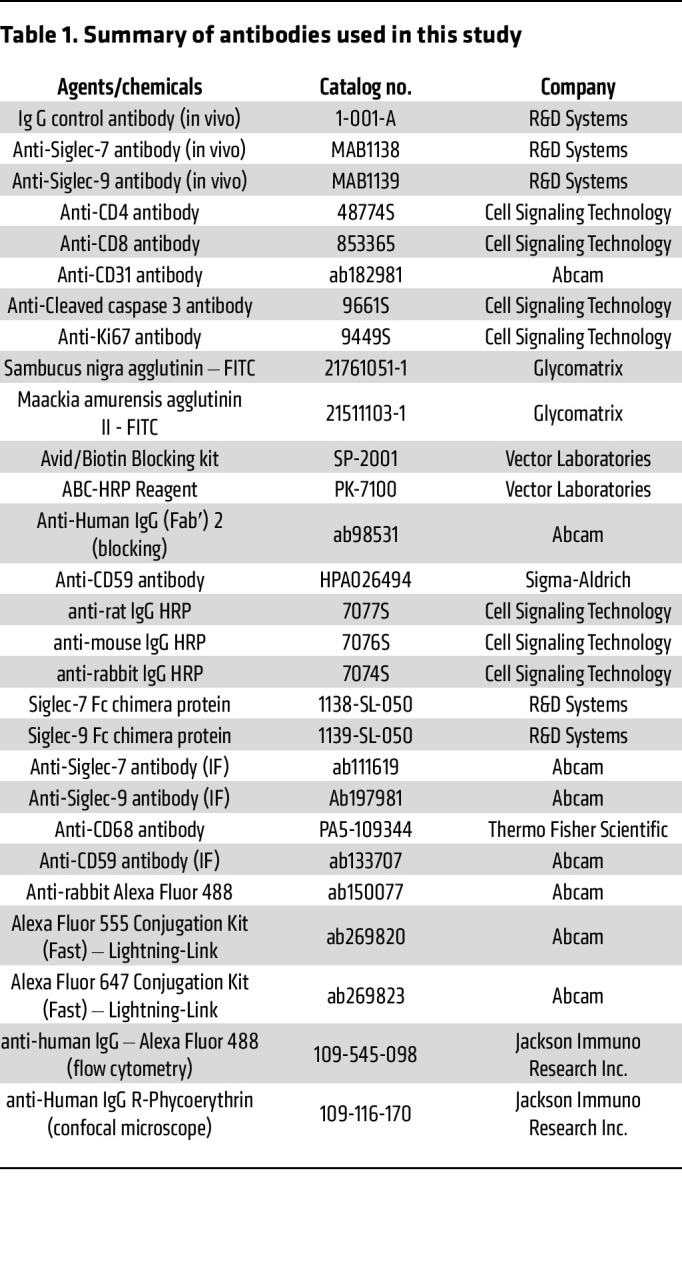
Summary of antibodies used in this study

## Version 1 10/22/2024

In-Press Preview

## Version 2 12/16/2024

Electronic publication
