# Supplementary material for: Sialylated glycoproteins suppress immune cell killing by binding to Siglec-7 and Siglec-9 in prostate cancer
Source: J Clin Invest. 2024 Oct 22;134(24):e180282. doi: 10.1172/JCI180282 (PMC11645153; doi:10.1172/JCI180282)
Supplement: Supplemental data [file jci-134-180282-s049.pdf]

Sialylated glycoproteins suppress immune cell killing by binding to Siglec-7 and Siglec-9 in prostate cancer

Ru M Wen[1*], Jessica C Stark[2,3], G. Edward W Marti[4], Zenghua Fan[5,6], Aram Lyu[5,6], Fernando Jose Garcia Marques[7,8], Xiangyue Zhang[9], Nicholas M Riley[3], Sarah M Totten[7,8], Abel Bermudez[7,8], Rosalie Nolley[1], Hongjuan Zhao[1], Lawrence Fong[5,6], Edgar G Engleman[9], Sharon J Pitteri[7,8], Carolyn R Bertozzi[2,10], James D Brooks[1,8*]

1 Department of Urology, Stanford University, Stanford, CA 94305, USA

2 *Department of Chemistry and Sarafan ChEM-H, Stanford University, Stanford, CA 94305,*

3 Department of Biological Engineering, Department of Chemical Engineering, Koch Institute for Integrative Cancer Research, Massachusetts Institute of Technology, Cambridge, MA 02139 , USA

4 Department of Molecular and Cellular Physiology, Stanford University, Stanford, CA 94305, USA

5 Department of Medicine, University of California San Francisco, San Francisco, CA, USA

6 Parker Institute for Cancer Immunotherapy, San Francisco, CA, USA

7 Department of Radiology, Stanford University, Stanford, CA 94305, USA

8 Canary Center at Stanford for Cancer Early Detection, Stanford University, Stanford, CA 94305, USA

9 Department of Pathology, Stanford University, Stanford, CA 94305, USA

10 Howard Hughes Medical Institute, Stanford, California, USA

Hematoxylin and Eosin staining

The tissue sections were stained with Hematoxylin and Eosin as described previously (1). Briefly, after baking, tissue slides were immersed in a series of solution including Clearify (SKU #: CACLELT, Statlab LLC, Texas TX), 100% ethanol, 95% ethanol and 75% ethanol for 5 min each time with 2 times each solution. The sections were stained with Ehrlich's Hematoxylin for 5 min, followed by quick dips (5-6 dips) in acid alcohol (0.3%), Scott's Tap Solution for 3 min, Eosin solution for 30 seconds with slides washed with tap water for 3 times. Dehydration was performed by immersing the sections in 95% alcohol, 100% alcohol, and xylene for 2 min each (2X). Finally, the sections were air dried for 10 min and mounted with a coverslip.

Immunohistochemistry staining

The tissue sections were stained with CD4, CD8, CD31, CD68, Ki67, and cleaved caspase 3 as described (1). The tissue sections were baked at 60 °C for 60 min and then immersed in a series of solvents: Clearify (5 min, 2X), 100% ethanol (5 min, 2X), 95% ethanol (5 min, 2X), and 70% ethanol (5 min, 1X). For antigen retrieval, the tissue sections were boiled in 0.01 M citrate buffer (pH 6.0) for 30 min, and slides were blocked in 5% goat serum and 5% BSA in PBS for 1 h. After that, the sections were incubated with a primary antibody (CD4, CD8, CD31, CD68, Ki67, and cleaved caspase 3) in PBS containing 0.5% BSA overnight at 4°C. The following day, the sections were washed with PBS (3X) and treated with 0.3% H_2O_2 in distilled water to block endogenous peroxidase activity. Subsequently, a secondary antibody was applied in PBS containing 0.05% BSA. DAB solution (catalog No. SK-4100) was used to develop the tissue slides. The slides were stained with Hematoxylin for 5 minutes, followed by immersion in $NaHCO_3$ solution for 3 minutes. The slides were dehydrated by immersing in 75%, 95%, and 100% ethanol, and xylene, for 5 min each (2X). The tissue sections were air dried and mounted with a coverslip.

Conjugation of antibody to fluorescent dyes

The anti-CD68 antibody was conjugated to Alexa Fluor 555 and anti-Siglec-9 antibody was conjugated to Alexa Fluor 647 using a lightning-link conjugation kit according to manufacture's instructions. Specifically, 1 µL of Modifier reagent was added into 10 µL of CD68 or Siglec-9 antibody. The antibody-modifier mix was added into a vial of Alexa Fluor® 555 or 647 Conjugation mix (10 µg), and incubated at room temperature for 3h in the dark. The reaction was stopped by adding Quencher reagent (1 µL) with gentle mixing for 30 min. The CD68-Alexa Fluor® 555 and Siglec-9-Alexa Fluor® 647 conjugates were stored at 4ºC.

Immunofluorescence staining

The tissue sections were stained with CD68, Siglec-7 and Siglec-9 as described previously (1). Briefly, slides were first incubated overnight with the primary antibody anti-Siglec-7 (catalog no. ab111619, Abcam, 1:200). After washing three times with TBST, the slides were incubated with the secondary antibody anti-rabbit Alexa Fluor 488 (catalog no. ab150077, Abcam), diluted 1:400 in 1× PBS containing 0.05% BSA, for 1 hour at room temperature. Following three washes with TBST, the slides were incubated with CD68-Alexa Fluor® 555 (1:200) and Siglec-9-Alexa Fluor® 647 (1:100) conjugates for 2 hour at room temperature. The slides were then washed three times with TBST, mounted using mounting medium with DAPI (catalog no. ab104139), and sealed with clear nail polish. The slides were imaged by confocal microscope (ZEISS LSM980).

T Cell Purification and Culture

CD8+ T cells were isolated from PBMCs using an EasySep™ Human CD8+ T Cell Isolation Kit (Catalog # 17953) following the manufacturer's instructions (STEMCELL Technologies). CD8+ T cells isolated by negative immunomagnetic selection were cultured in RPMI 1640 medium (Gibco), supplemented with 10% fetal bovine serum (FBS) (Thermo Fisher Scientific) and 1%

Penicillin/Streptomycin (Thermo Fisher Scientific). For activation and expansion, CD8+ T cells were cultured in the presence of anti-CD3/CD28 beads (catalog no 11131D, Life Technologies) in RPMI medium containing IL-2 (2 ng/mL) for 4 days.

Cytotoxicity measurement

Target cell lines including PC3, DU145, LNCaP were labelled with GFP using a lentiviral system and plated into 96-well plates. Subsequently, activated CD8+ T cells were added to the target cells at a ratio of 1:10 (T:E) and incubated for 16 hours. The medium containing T cells in suspension and dead target cells was removed, and lysis buffer was added to the plate. GFP fluorescence of the target cells was measured using a plate reader with excitation at 480 nm and emission detection at 525 nm. A standard curve of cell numbers was established to quantify the cytotoxicity of CD8+ T cells based on the measured GFP fluorescence intensity.

Transcriptome data analysis

For the correlation matrix analysis, the procedure and curated list of 531 immune genes and 34 genes related to sialoglycan biosynthesis (SBGs) from Stanczak, et al was largely adapted to the TCGA dataset(2). After excluding 4 overlapping between immune genes and SBGs, as well as 30 immune genes and one SBG not detected in the PRAD-US dataset, the analysis was carried out the remaining 497 immune genes and 33 SBGs. The values of the correlation matrix are the Pearson correlation coefficient between the log normalized expression of each immune gene with each SBG across all primary tumor samples, where normalization is log_10 (1 + transcripts per million). K-means clustering of Principal Component Analysis (PCA) (sklearn.decomposition.PCA and sklearn.cluster.KMeans) and hierarchical/agglomerative clustering (sklearn.cluster.AgglomerativeClustering) were applied to cluster SBGs and yielded identical clusters for k=3. Gene Ontology (GO) term enrichment analysis was performed with

g:Profiler on immune genes showing significant Pearson correlation variations between SBG clusters (statistical domain scope is set to all 497 immune genes). Significance was determined for each immune gene, and in each cluster, by a t-test between the mean Pearson correlation coefficient with SBGs in that cluster against SGBs in all other clusters (scipy.stats.ttest_ind), adjusted by the Benjamini-Hochberg procedure (alpha=0.01).

Glycopeptide Identification

Byonic™ Software by Protein Metrics (version v2.11.0, Cupertino, CA) was used for glycopeptide identification. Each file was searched against the curated prostate proteomic database with added decoys, and a custom glycan database containing 331 human glycan compositions. Search parameters were set as follows: precursor mass tolerance of 10 ppm (where precursor mass and charge assignments were computed from the MS1), fragment mass tolerance of 20 ppm, fixed carbamidomethyl modification at cysteine, common modifications of oxidation at methionine, glycosylation at asparagine given consensus sequence N-X-S/T (where X is any amino acid excluding proline), and a 1% protein FDR cutoff. Glycopeptides from the Byonic search results that met the criteria of having a Score > 150 and Log Probability > 1.5 were retained for further analysis. Only proteins that were associated with two or more glycopeptides were retained for further analysis.

Intact Glycoproteomics Data Analysis

In our glycoproteomic analysis, we addressed the challenge of misidentifying glycopeptides due to the minimal mass difference between two fucoses and one sialic acid. Our method involved a three-step correction process: first, selecting glycopeptide identifications with at least two fucoses and a mass accuracy greater than -1 Da; second, calculating the maximum number of sialic acids based on the number of hexoses minus three; and third, replacing two fucoses with one sialic acid

in identifications where the actual sialic acid count was below the maximum. For classification, we categorized glycopeptides into seven types based on sugar composition, including high mannose, hybrid, and various complex types, determined by the counts of HexNAc and Hex. Finally, we quantified each identified glycoprotein according to these classifications, normalizing the spectral counts per protein and glycan type relative to the total spectral counts for each glycoprotein, to ensure accurate analysis.

Supplemental Table S1 A summary of the chemical agents and antibodies

**Agents/chemicals**	**Catalog no**	**Company**
FBS	26140079	Life Technologies
DNase I	10104159001	Sigma
Collagenase IV	LS0004186	Worthington Biochemical
EDTA	1233508	Sigma
Neuraminidase (sialidase)	P0722L	NEB
Laemmli Sample Buffer	#1610747	Bio-rad
FBS	FB-01	Omega Scientific, Inc.
Accutase	AT104	Innovative Cell Technologies, Inc
Dynabeads™ Protein G	10003D	ThermoFisher Scientific
Dynabeads™ Human T-Activator CD3/CD28	11131D	ThermoFisher Scientific
Halt™ Protease Inhibitor Cocktail	87786	ThermoFisher Scientific
RIPA buffer	89900	ThermoFisher Scientific
IP lysis buffer	87788	ThermoFisher Scientific
BCA protein assay kit	23225	ThermoFisher Scientific
GM-CSF	572914	Biolegend
10% NP-40	ab142227	Abcam
Rapigest	186001860	Waters
Triethylammonium bicarbonate buffer	60044973	Fluka
Iodoacetamide	A3221-10VL;	Sigma
Formic acid	28905	Fisher
Strata-X columns	8B-S100-AAK	Phenomenex
IL-2	589102	Biolegend
CRISPRi-v2	1000000090	Addgene
dCas-9-KRAB	46911	Addgene
CD59 sgRNA CRISPR/Cas9	15555111	Applied Biological Materials Inc.
CRISPR Scrambled sgRNA	K010	Applied Biological Materials Inc.
pLV-C-GFPSpark	LVCV-35	Sino Biological Inc
Compensation Beads	01-3333-41	Invitrogen
Sialic acid assay kit	ab83375	Abcam
Nucleaspin blood XL for DNA	740950.50	MACHEREY-NAGEL
Plasmid DNA Maxi Kit	D6924-03	Omega Bio-Tek
DAB solution	SK-4100)	Dako
dual Endogenous Enzyme Block	S2003	Dako, Agilent
anti-human lgG HRP	98595	Abcam
Anti-rat lgG	BP9400	Vector Laborateries
anti-mouse lgG	BP9200	Vector Laborateries

anti-rabbit lgG	BP9100	Vector Laborateries
anti-rat lgG HRP	7077S	Cell Signaling Technology
Anti-Rabbit IgG (Light-Chain Specific)	93702S	Cell Signaling Technology
Anti-rabbit IgG (Conformation Specific)	5127S	Cell Signaling Technology

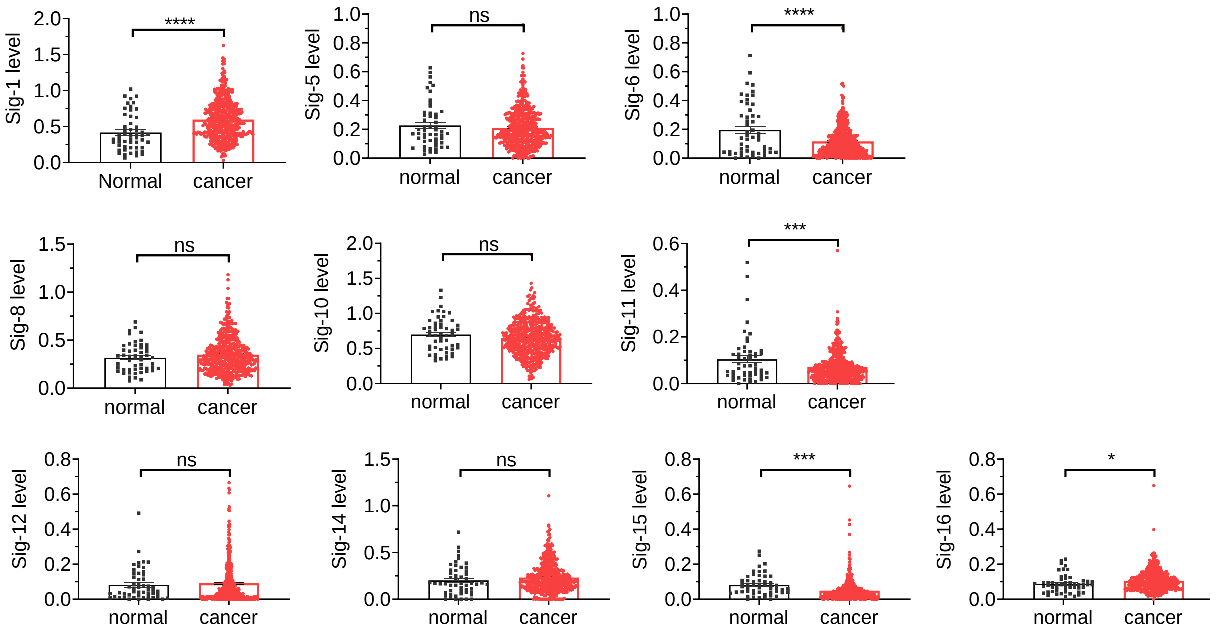

Supplemental Figure S1: The expression of Siglec isoforms in prostate cancer from the PRAD-TCGA database.

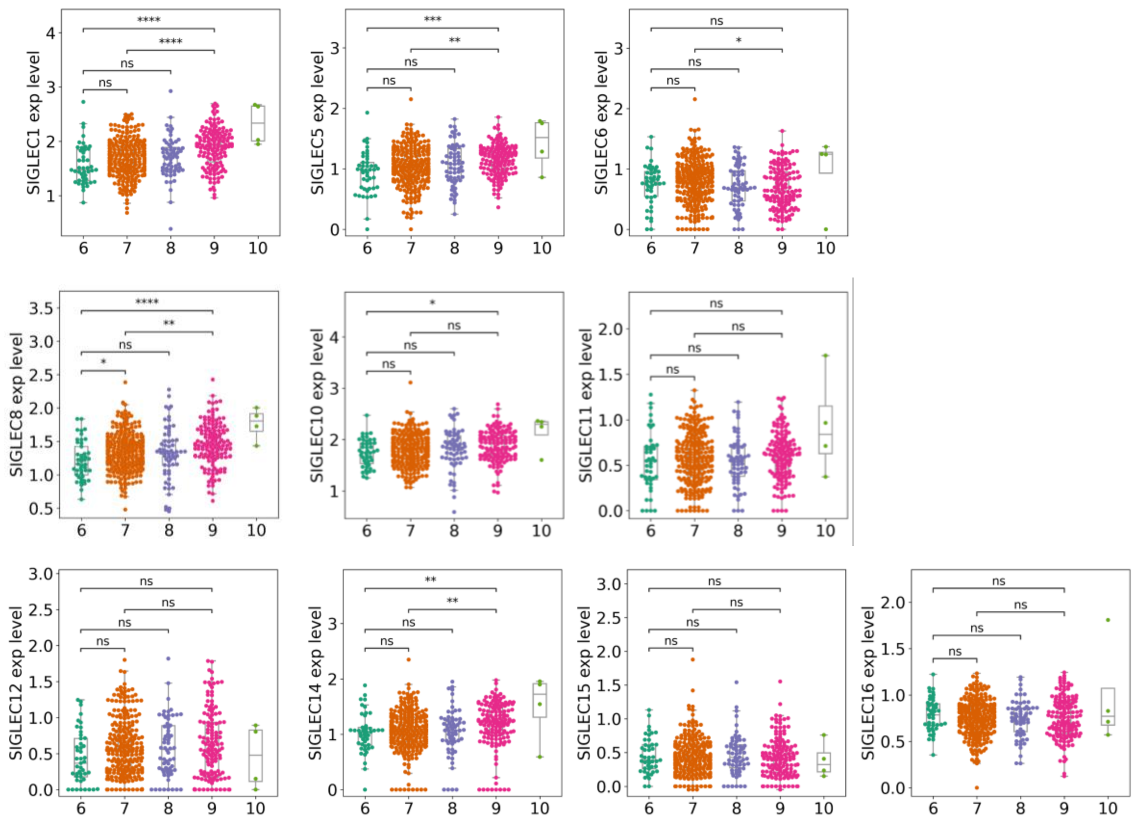

Supplemental Figure S2: The correlation of Siglec isoform expression with Gleason Score from the PRAD-TCGA database.

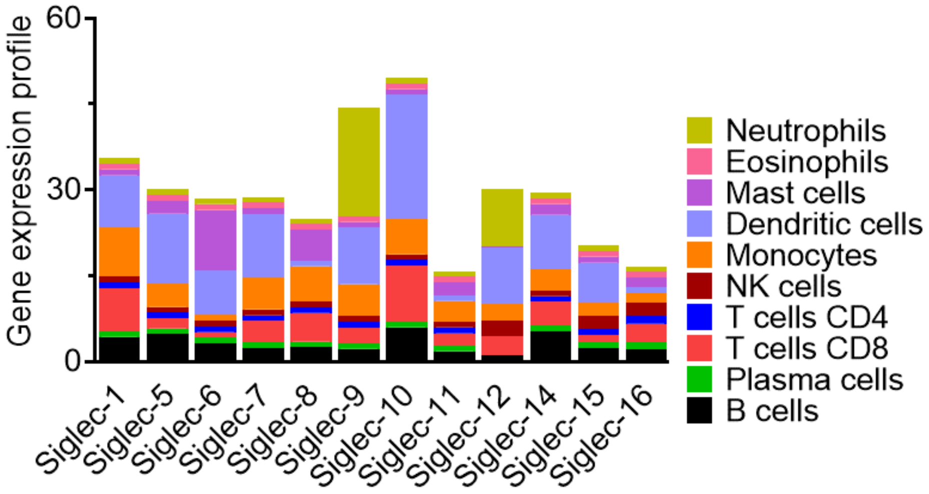

Supplemental Figure S3: Gene expression profile of Siglecs in prostate cancer tumor-infiltrating immune cells identified using CIBERSORTx from the PRAD-TCGA database.

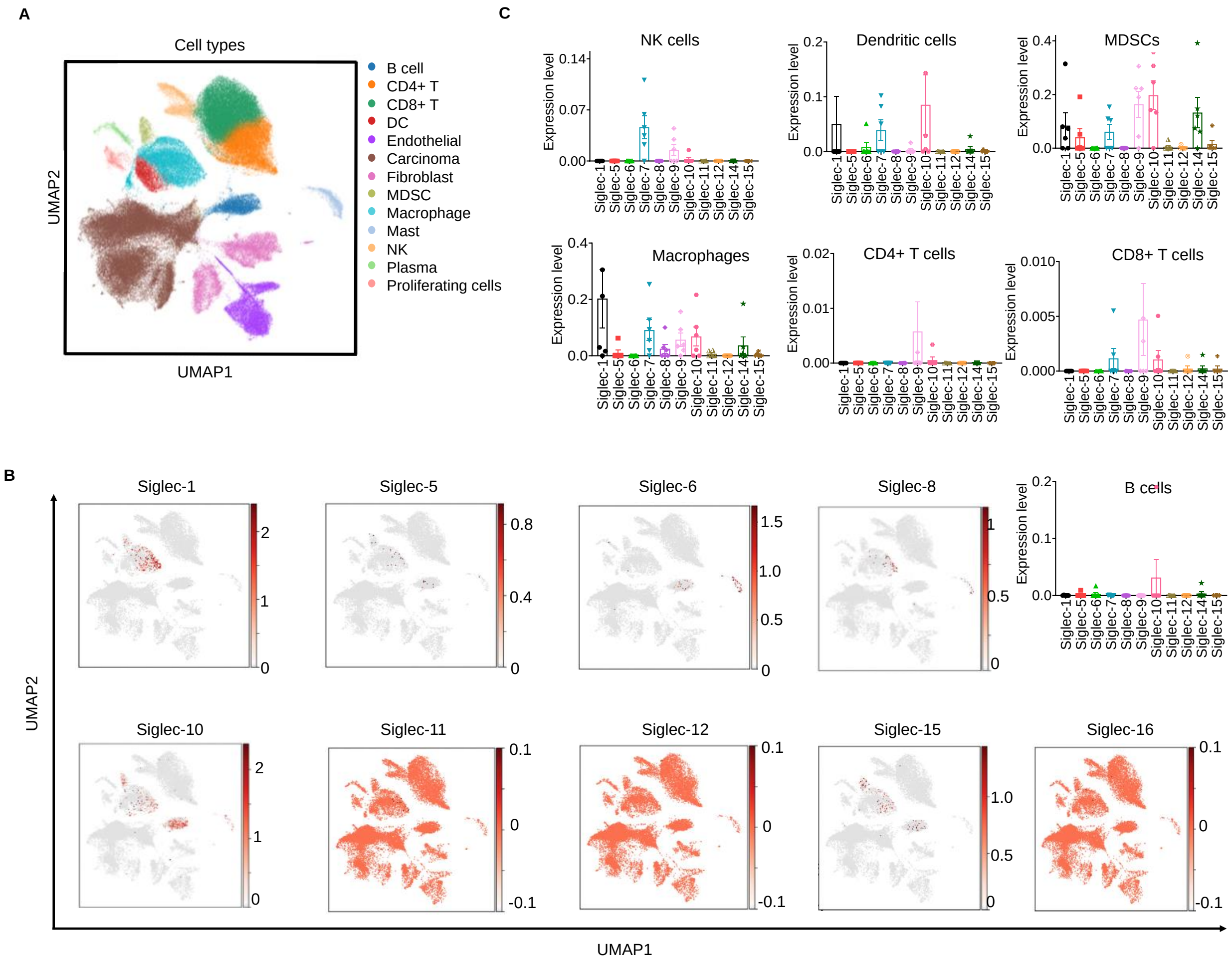

Supplemental Figure S4: Single-cell RNA sequencing analysis of Siglec expression profiles in human metastatic castration-resistant prostate cancer (CRPC) tumors. (A) The UMAP plot displays Siglec expression across immune cells within CRPC tumors. (B) The expression levels of Siglecs in NK cells, dendritic cells, CD4^{+} T cells, and CD8^{+} T cells. Siglec-1, -5, -10, and -15 are primarily expressed on macrophages and myeloid-derived suppressor cells (MDSCs). Additionally, Siglec-10 is expressed on B cells and mast cells, while Siglec-15 is also expressed on B cells. Siglec-6 is expressed on B cells and mast cells.

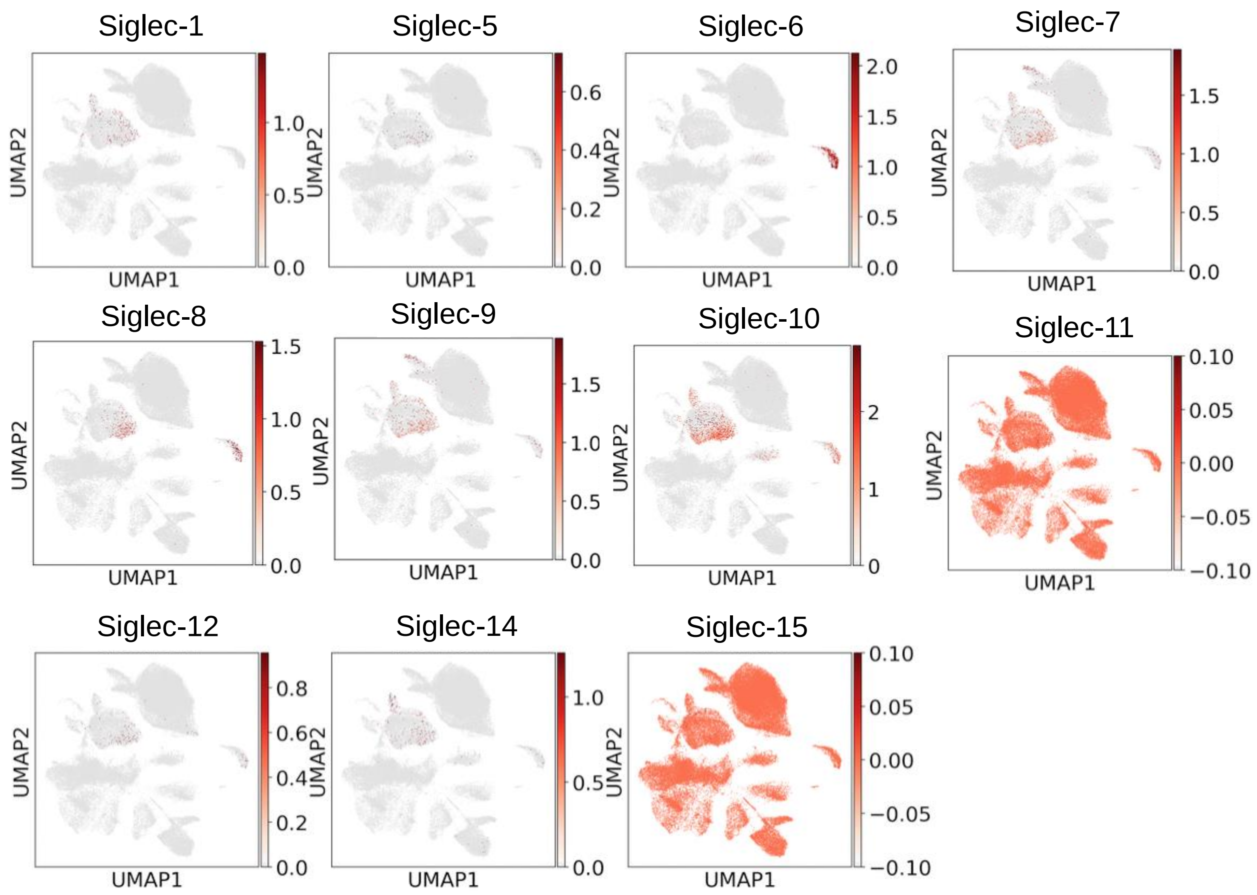

Supplemental Figure S5: Single-cell RNA sequencing analysis of Siglec expression profiles in human localized prostate cancer tumors. Siglec-1, -5, -7, -9, -10, and -14 are predominantly expressed on macrophages and myeloid-derived suppressor cells (MDSCs). Additionally, Siglec-10 is also expressed on B cells and mast cells, while Siglec-7 and -9 are expressed on mast cells. Siglec-6 is primarily expressed on mast cells, and Siglec-8 is mainly expressed on both macrophages and mast cells.

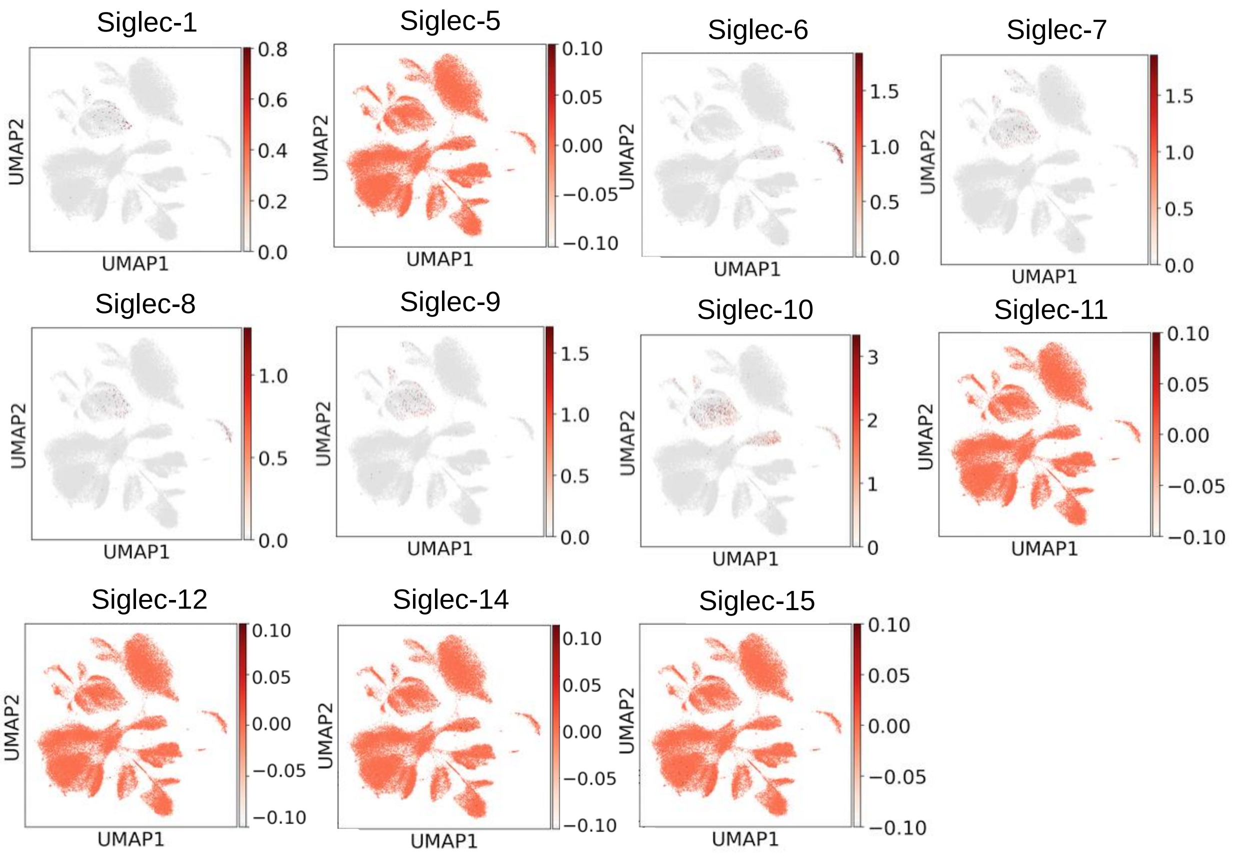

Supplemental Figure S6: Single-cell RNA sequencing analysis of Siglec expression profiles in human metastatic hormone-sensitive prostate cancer tumors. Siglec-7, -9, and -10 are primarily expressed on macrophages and myeloid-derived suppressor cells (MDSCs). Siglec-10 is mainly expressed on B cells and mast cells. Siglec-1 is predominantly expressed on macrophages, while Siglec-6 is mainly expressed on mast cells. Siglec-8 is primarily expressed on both macrophages and mast cells.

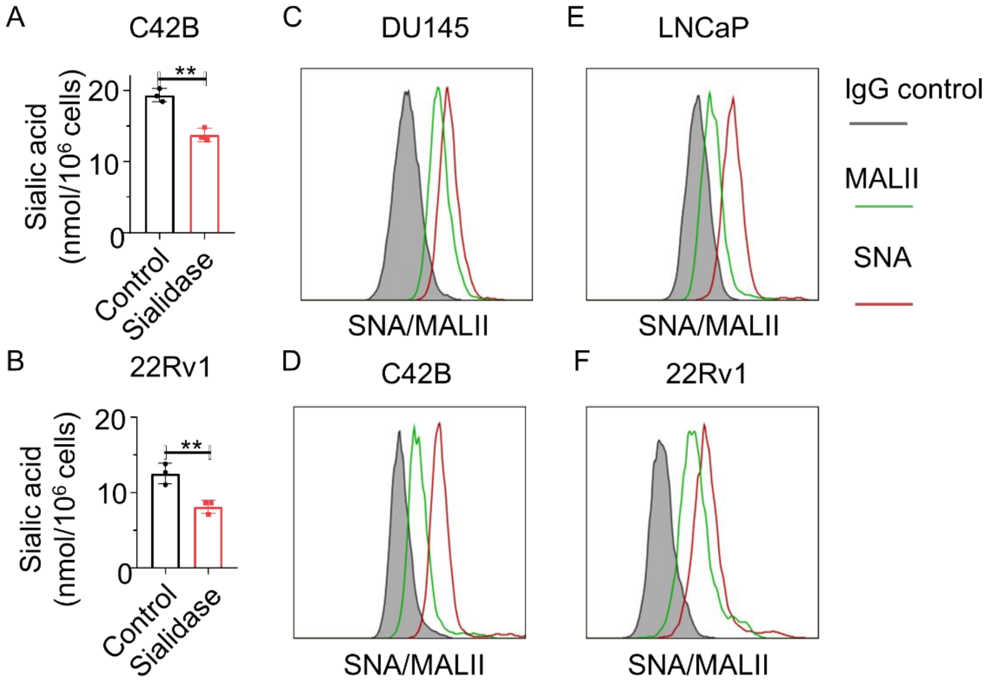

Supplemental Figure S7: Surface sialic acid concentration in (A) C42B cells, and (B) 22Rv1 cells. The expression of SNA and MALII in (C)DU145, (D) C42B, (E) LNCaP, and (F) 22Rv1 cells.

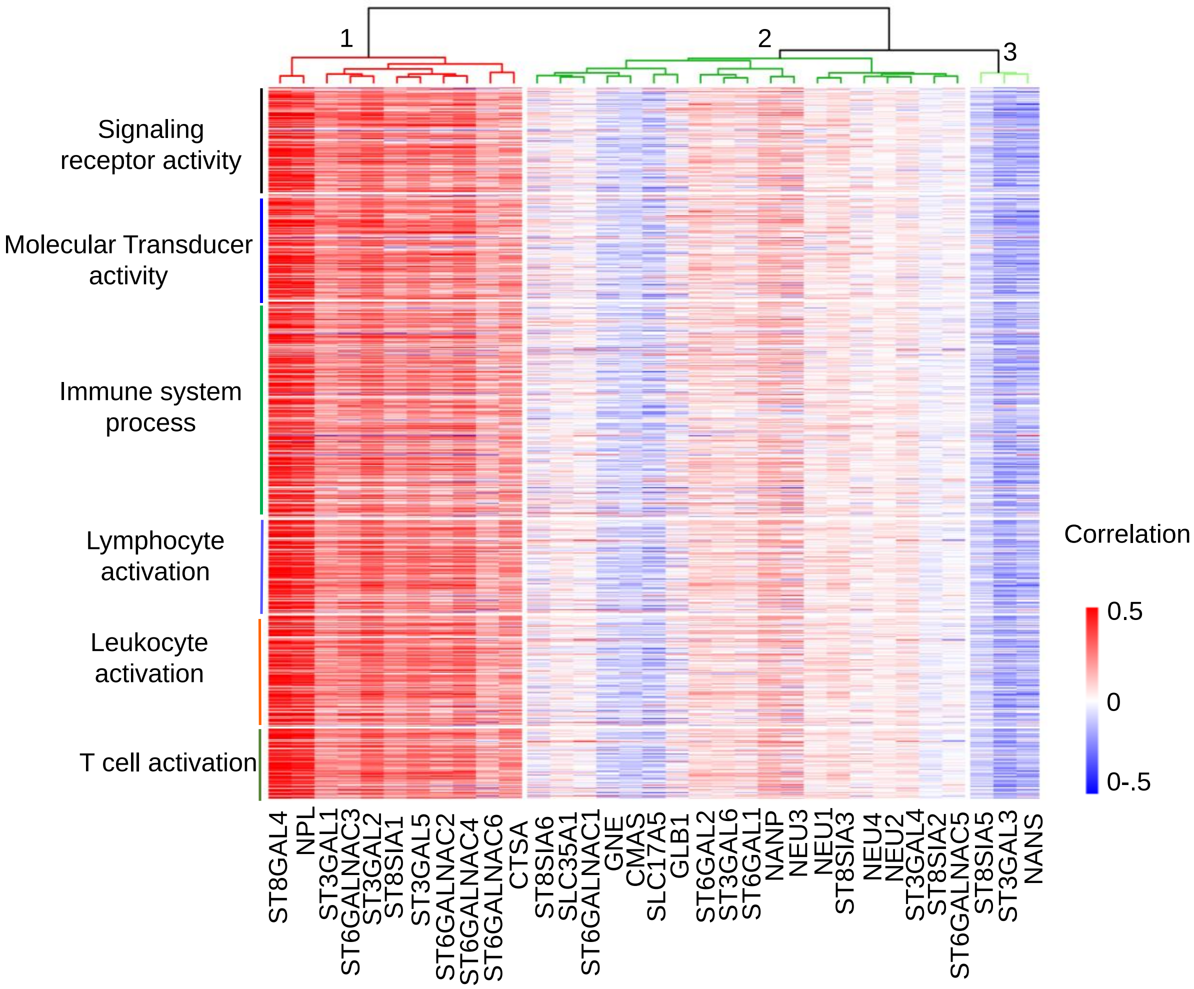

Supplemental Figure S8: K-means clustering analysis of correlation between expression of sialoglycan biosynthetic genes and 497 immune genes the PRAD-TCGA database. Each row corresponds to an immune cell gene, and each column corresponds to a sialoglycan gene. GO-term analysis was used to group immune genes into functional classes. Red indicates positive correlation between expression of the sialoglycan gene and the level of expression of the immune gene across all patients in PRAD-TCGA, while blue indicates an inverse correlation. Unsupervised clustering identifies a set of sialoglycan biosynthetic genes with strong correlation to immune gene expression, and two additional subclasses with equivocal correlation.

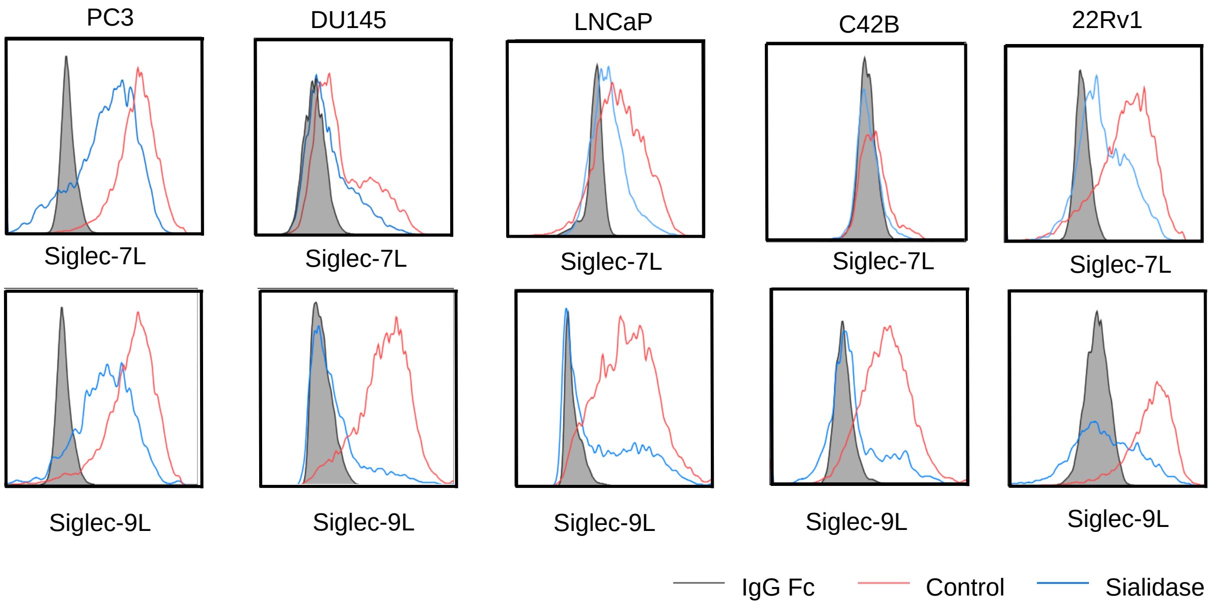

Supplemental Figure S9: The expression of Siglec-7 ligands (Siglec-7L) and Siglec-9 ligands (Siglec-9L) in PC3, DU145, LNCaP, C42B, and 22Rv1 cells upon sialidase treatment by flow cytometry. The experiments were completed with different settings to best demonstrate the efficacy of sialidase treatment, and therefore, they are not suitable for direct comparison between cell lines.

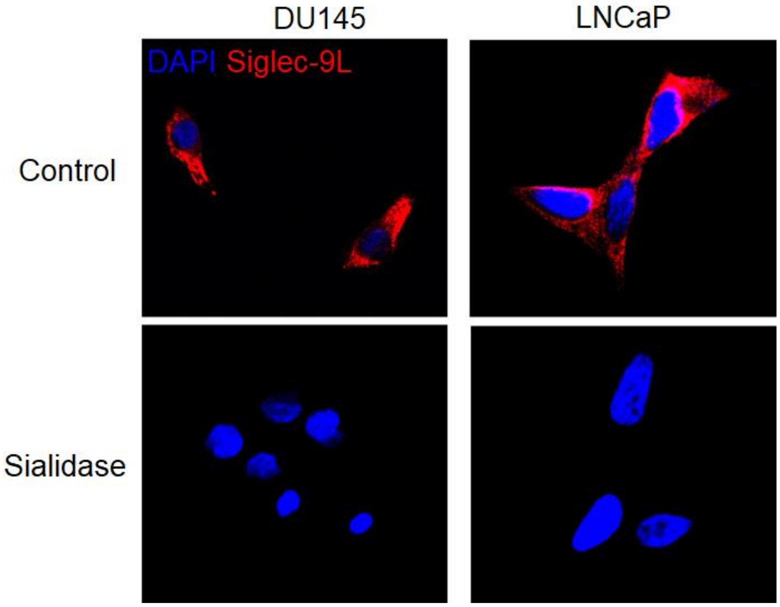

Supplemental Figure S10: Representative image of Siglec-9-Fc immunofluorescent staining of DU145 and LNCaP cells by confocal microscopy.

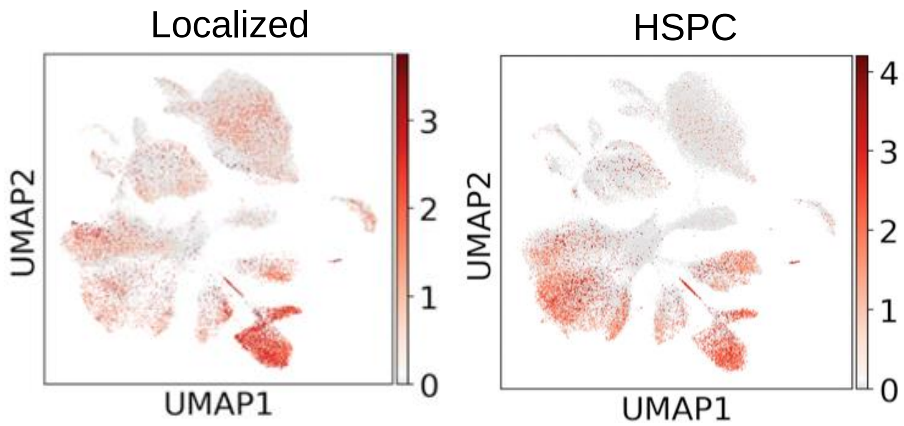

Supplemental Figure S11: Single-cell RNA sequencing analysis of CD59 expression profiles in human localized and metastatic hormone-sensitive prostate cancer (HSPC) tumors. Refer to Supplementary Figure S4 for cell type information. CD59 is expressed on tumor cells in both localized and metastatic HSPC tumor tissues.

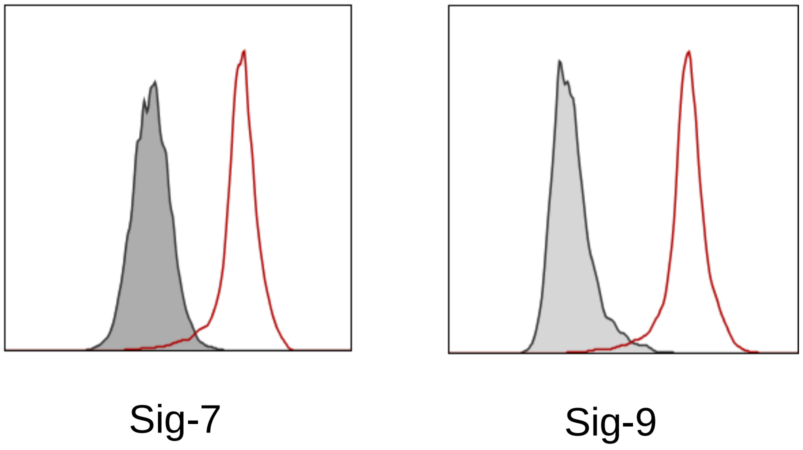

Supplemental Figure S12: Flow cytometry analysis reveals that the activated CD8^{+} T cells used in this study express Siglec-7 and Siglec-9.

Reference

1. Wen RM, Qiu Z, Marti GEW, Peterson EE, Marques FJG, Bermudez A, et al. AZGP1 deficiency promotes angiogenesis in prostate cancer. *Journal of translational medicine.* 2024;22(1):383.
2. Stanczak MA, Rodrigues Mantuano N, Kirchhammer N, Sanin DE, Jacob F, Coelho R, et al. Targeting cancer glycosylation repolarizes tumor-associated macrophages allowing effective immune checkpoint blockade. *Sci Transl Med.* 2022;14(669):eabj1270.
