# Supplementary figures and images for: Sialylated glycoproteins suppress immune cell killing by binding to Siglec-7 and Siglec-9 in prostate cancer
Source: J Clin Invest. 2024 Oct 22;134(24):e180282. doi: 10.1172/JCI180282 (PMC11645153; doi:10.1172/JCI180282)

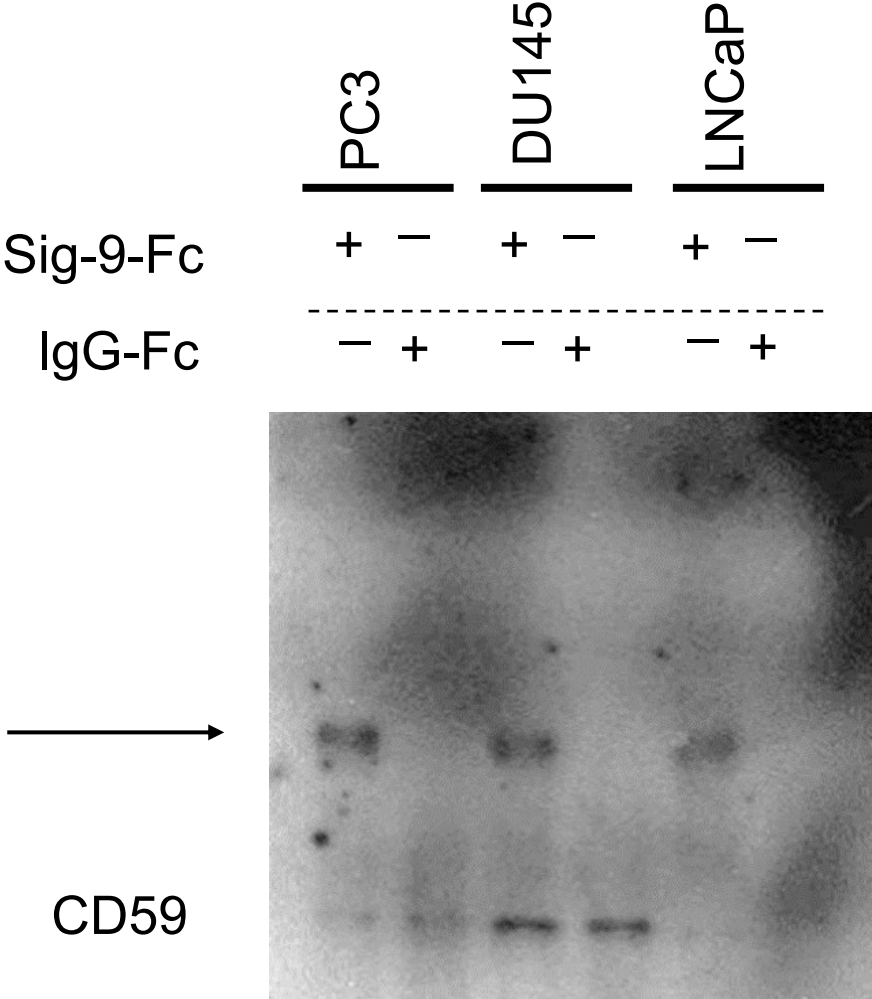

Full unedited gel for Figure 6F

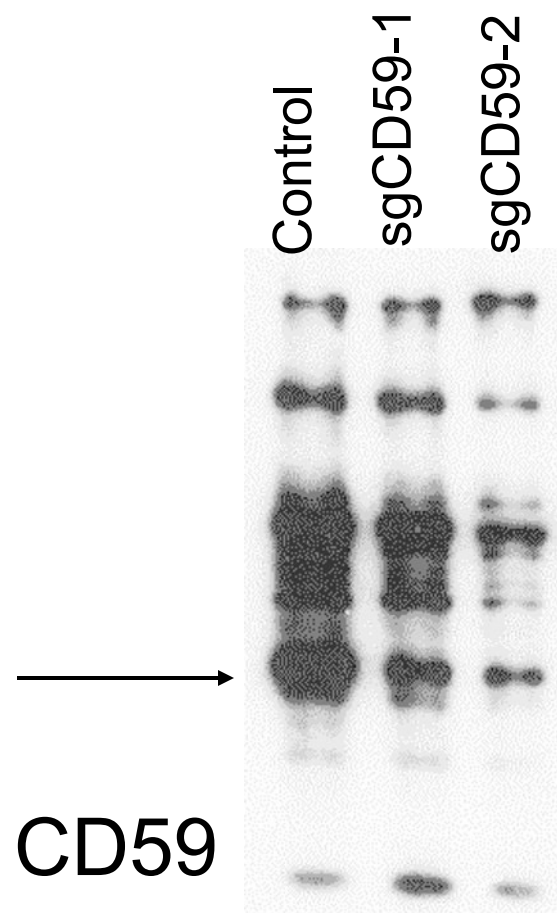

Full unedited gel for Figure 6G

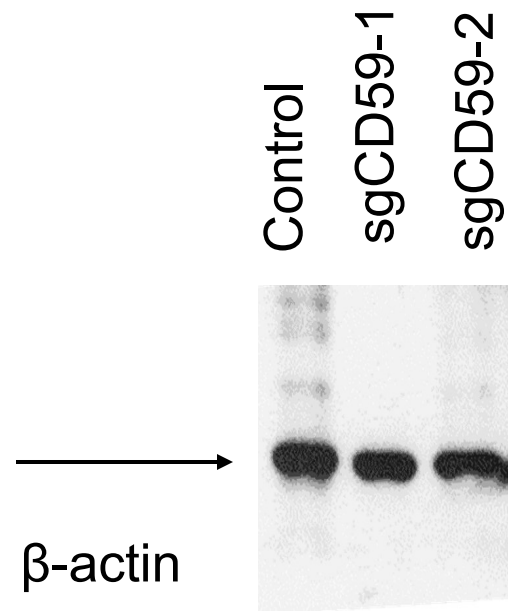

Full unedited gel for Figure 6G

Supplement: Unedited blot and gel images [file jci-134-180282-s050.pdf]
